# Electronic and Structural Properties of Thin Iron Oxide Films on CeO_2_

**DOI:** 10.1021/acsami.4c05542

**Published:** 2024-08-21

**Authors:** Lesia Piliai, Pablo Castro-Latorre, František Pchálek, Shiva Oveysipoor, Yuliia Kosto, Ivan Khalakhan, Tomáš Skála, Konstantin M. Neyman, Pere Alemany, Michael Vorochta, Albert Bruix, Peter Matvija, Iva Matolínová

**Affiliations:** † Department of Surface and Plasma Science, Faculty of Mathematics and Physics, Charles University, V Holešovičkách 2, Prague 8 180 00, Czech Republic; ‡ Departament de Ciència de Materials i Química Física and Institut de Química Teòrica i Computacional (IQTCUB), Universitat de Barcelona, Barcelona 08028, Spain; § Applied Physics and Semiconductor Spectroscopy, Brandenburg University of Technology Cottbus-Senftenberg, Konrad-Zuse-Strasse 1, Cottbus 03046, Germany; ∥ ICREA (Institució Catalana de Recerca i Estudis Avançats), Barcelona 08010, Spain

**Keywords:** ceria, CeO_2_, iron oxide, 2D layer, catalysis, STM, XPS, DFT

## Abstract

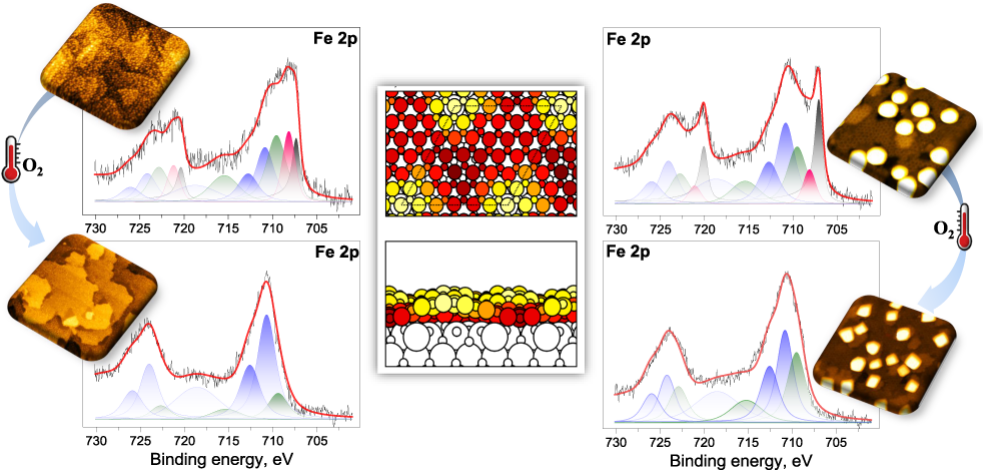

Modification of CeO_2_ (ceria) with 3d transition metals, particularly iron, has been proven to significantly enhance its catalytic efficiency in oxidation or combustion reactions. Although this phenomenon is widely reported, the nature of the iron–ceria interaction responsible for this improvement remains debated. To address this issue, we prepared well-defined model FeO_*x*_/CeO_2_(111) catalytic systems and studied their structure and interfacial electronic properties using photoelectron spectroscopy, scanning tunneling microscopy, and low-energy electron diffraction, coupled with density functional theory (DFT) calculations. Our results show that under ultrahigh vacuum conditions, Fe deposition leads to the formation of small FeO_*x*_ clusters on the ceria surface. Subsequent annealing results in the growth of large amorphous FeO_*x*_ particles and a 2D FeO_*x*_ layer. Annealing in an oxygen-rich atmosphere further oxidizes iron up to the Fe^3+^ state and improves the crystallinity of both the 2D layer and the 3D particles. Our DFT calculations indicate that the 2D FeO_*x*_ layer interacts strongly with the ceria surface, exhibiting structural corrugations and transferred electrons between Fe^2+^/Fe^3+^ and Ce^4+^/Ce^3+^ redox pairs. The novel 2D FeO_*x*_/CeO_2_(111) phase may explain the enhancement of the catalytic properties of CeO_2_ by iron. Moreover, the corrugated 2D FeO_*x*_ layer can serve as a template for the ordered nucleation of other catalytically active metals, in which the redox properties of the 2D FeO_*x*_/CeO_2_(111) system are exploited to modulate the charge of the supported metals.

## 1 Introduction

Over the past decade, cerium dioxide (ceria, CeO_2_) has remained a leading material for energy storage, electrocatalysis, and heterogeneous catalytic applications.^1^ Benefiting from the unique redox potential of the Ce^4+^–Ce^3+^ couple and the facile formation of oxygen vacancies (V_O_), ceria is able to provide an excellent support for noble metals which are known to exhibit high catalytic performance.^2^ Although noble metal-based catalysts show sufficient activity over a wide range of temperatures, their scale-up implementation is limited by their scarcity and high cost. Therefore, the development of an active and inexpensive catalyst is highly desirable.

Modification of CeO_2_ with transition metals (TMs) such as iron aims to enhance its catalytic performance while keeping the price of the resulting catalysts low.^3^ Recent studies have shown that the addition of iron to ceria significantly improves its performance in CO oxidation, NO oxidation and soot combustion.^3−5^ Morphologically, this can be attributed to an increased specific surface area and a higher number of open active sites.^4,6^ Electronically, it has been suggested that the promotion of Ce-based oxides with Fe species facilitates electron transfer between the mixed Fe^2+^–Ce^4+^ and Fe^3+^–Ce^3+^ sites, which had been identified as active centers in various catalytic processes.^6,7^ Furthermore, iron-modified ceria shows not only improved redox properties but also promotes the dispersion of supported metal nanoparticles and enhances the interaction of metal species with ceria supports.^8^

While the improved activity of Fe–Ce mixed oxide catalysts has been widely reported, the nature of the iron–ceria interaction remains controversial. First, many studies have shown that the introduction of redox-active cations into the CeO_2_ lattice gives rise to the formation of homogeneous solid solutions.^8,9^ Specifically, the presence of Fe dopant leads to strong structural distortions, resulting in a hematite-like mixed oxide^10^ or ceria-like solid solutions^9^ with a higher density of oxygen vacancies at the surface. However, findings presented by Polychronopoulou^11^ and others^12,13^ demonstrate that iron has a poor solubility in the ceria fluorite lattice compared to the rest of binary TM-doped (TM = Cu, Co, Ni, Zn) ceria systems. Second, it has been documented that the doping of low valence cations such as Fe^3+^ and Fe^2+^ in CeO_2_ can facilitate the formation of surface oxygen vacancies.^4,7,14^ The increased occurrence of oxygen vacancies in the Ce–Fe mixed oxide system can be attributed to the combined redox behavior of cerium (Ce^4+^/Ce^3+^) and iron (Fe^3+^/Fe^2+^) cations.^14^ However, Li et al. reported that the distribution of Fe^3+^ over Ce^4+^ sites leads to the absence of Ce^3+^ ions and consequently results in a low oxygen vacancy concentration.^9^ The absence of Ce^3+^ ions is also supported by theoretical calculations, which show that Fe adsorption on the stoichiometric CeO_2_(111) surface suppresses the formation of oxygen vacancies in the ceria lattice.^15^ These findings underscore the necessity for a better understanding of the Fe–CeO_2_ interaction.

In contrast to the majority of previous research, which has primarily focused on complex powder catalysts investigating parameters such as iron content^16^ and the distribution of iron ions^17^ in Fe-doped CeO_2_ structures, the present study examines more precisely defined single-crystalline systems. Despite not having exactly the same characteristics as technical catalysts based on combinations of ceria and Fe, our model systems exhibit many of the surface sites defining the catalytic properties of mixed FeO_*x*_–CeO_2_ systems: bare CeO_2_(111) facets, CeO_2_(111) step edges, FeO_*x*_ 3D clusters, the FeO_*x*_ 2D layer with edges, and the FeO_*x*_–CeO_2_ interface. We investigate the electronic and chemical states of these features using synchrotron radiation photoelectron spectroscopy (SRPES), resonant photoelectron spectroscopy (RPES) and conventional X-ray photoelectron spectroscopy (XPS) techniques. We explore the thermally induced morphological and structural changes of FeO_*x*_/CeO_2_(111), particularly the formation of a thin FeO_*x*_ layer over the ceria surface, through scanning tunneling microscopy (STM) and low-energy electron diffraction (LEED) techniques. To complement and further rationalize these experimental methods, we model the FeO_*x*_/CeO_2_(111) interface using density functional theory (DFT) calculations, focusing on the structure of the FeO_*x*_ thin film and the interaction between Fe^2+^/Fe^3+^ and Ce^4+^/Ce^3+^ redox pairs. Building upon our previous work on metal nanoparticles supported by CeO_2_(111) thin films,^18−20^ this multimodal approach not only allows us to gain valuable insights into the interactions within the FeO_*x*_–CeO_2_ system but also provides a deeper understanding of the structure–property relationship at the nanoscale.

## 2 Experimental Section

### 2.1 Sample Preparation

Well-ordered CeO_2_(111) thin films were prepared on Cu(111) and Pt(111) single crystals by the physical vapor deposition (PVD) technique. The Cu(111) (MaTecK GmbH, 99.999% purity) and Pt(111) (MaTecK GmbH, 99.99% purity) samples were cleaned by Ar^+^ sputtering at 300 K and annealing (750 and 950 K, respectively) until no traces of carbon or any other contaminants were found in the subsequent XPS analysis. The CeO_2_ thin films were grown by PVD of metallic Ce (Goodfellow GmbH, 99.9%) using an electron-beam evaporator (Tectra GmbH) in 5 × 10^–7^ mbar of oxygen at the deposition rate of about 0.1 monolayer (ML)/min, where 1 ML(CeO_2_(111)) corresponds to a 0.31 nm thick layer or about 7.9 × 10^14^ cm^–2^ of O–Ce–O groups. The substrate temperature during the CeO_2_(111) film growth was kept within a range of 523–723 K, depending on the desired density of step edges on the surface.^21^ The temperature was monitored by a K-type thermocouple attached to the back of the crystal. The thickness of the prepared layer was determined from the attenuation of the Cu 2p_3/2_ peak or the Pt 4f_7/2_ peak and was about 2.5 nm (8 ML of CeO_2_(111)). The surface structure of the CeO_2_ layer was confirmed by LEED. Fe (Goodfellow GmbH, 99.99%) was deposited by PVD from an electron-beam evaporator from an Fe rod (2 mm in diameter) onto the CeO_2_(111) surface. The deposition was carried out either stepwise (in the study of the growth mechanism) or in one deposition step (in the thermal stability study). The sample was grounded during the deposition. The nominal Fe thickness in Fe/CeO_2_(111) systems was determined from the attenuation of the Cu 2p_3/2_ signal from the Cu(111) substrate or the Pt 4f_7/2_ signal from the Pt(111) substrate. The thickness varied within the 0.3–2 ML range. Here, 1 ML = 1.72 × 10^15^ cm^–2^, which corresponds to a surface density of Fe atoms in the most stable (110) crystal plane in the Fe bcc crystal. In the case of STM measurements of the CeO_2_(111) surface covered exclusively by the 2D FeO layer, the amount of deposited Fe atoms was determined by considering the fraction of the surface covered by the FeO layer and its measured lattice parameter *a*_FeO_ = 0.31 nm.

### 2.2 Synchrotron Radiation Photoelectron Spectroscopy

High-resolution SRPES and resonant photoemission spectroscopy (RPES) experiments were carried out at the Materials Science Beamline (MSB) at the Elettra synchrotron light facility in Trieste, Italy. The UHV end-station of MSB with base pressure 2 × 10^–10^ mbar was equipped with a multichannel electron energy analyzer (Specs Phoibos 150), a nonmonochromatized Mg Kα X-ray source (1253.6 eV), LEED optics, a sputter gun (Ar^+^), and a gas inlet system for O_2_. During the experiment, two electron-beam evaporators for the deposition of Ce and Fe metals were used.

Spectra acquisition utilizing SRPES was performed for the Ce 4d, Fe 3p, C 1s, and O 1s core levels with photon energies of 180 eV (Ce 4d and Fe 3p), 410 eV (C 1s) and 650 eV (O 1s) to keep the electron kinetic energy around 100 eV with a total resolution of about 0.5 eV. RPES analysis was done by collecting the valence band (VB) spectra at 115, 121.4, and 124.8 eV photon energies. Ce^3+^ valence states resonate strongly at a photon energy of 121.4 resulting in a distinctive peak at about 1.4 eV. On the other hand, Ce^4+^ valence states reach their maximum at 124.8 eV, with a peak at about 4.0 eV. By subtracting the off-resonant spectra (115 eV) from the resonant spectra, it is possible to determine the features of D(Ce^3+^) and D(Ce^4+^) as shown in Supporting Information. The resonant enhancement ratio (RER) which is equal to D(Ce^3+^)/D(Ce^4+^) ratio, provides information about reduction state of the surface cerium cations.^18^ In addition to collecting the SRPES spectra, XPS spectra of O 1s, Ce 3d, Fe 2p, and Cu 2p_3/2_ core levels were acquired with a total resolution of 1 eV. All spectra were recorded at normal emission (SRPES and RPES) and 20° off normal (XPS). The binding energies in the spectra obtained with synchrotron radiation were calibrated with respect to the Fermi level (*E*_F_) measured on a clean gold foil. All PES data were processed using the KolXPD fitting software and normalized to the incident photon intensity, as determined by a flux monitor. The obtained Fe 3p and Fe 2p spectra were fitted after subtraction of a composite background which consisted of a baseline spectrum acquired before Fe deposition and the Shirley background. The results of XPS Fe 2p spectra fitting for metallic Fe, Fe_2_O, FeO and Fe_2_O_3_ are reported in Table S1.

### 2.3 Scanning Tunneling Microscopy

STM experiments were performed in a UHV system (base pressure 1 × 10^–10^ mbar) at Charles University, Prague, Czech Republic. The system is equipped with a scanning probe microscope (Specs SPM Aarhus 150 NAP), a photoelectron spectrometer consisting of a hemispherical electron energy analyzer with a 1D line detector (Specs Phoibos 150 1D-DLD) and a monochromatized Al Kα X-ray source (μ-FOCUS 600 equipped with XR 50 MF), LEED, and a quadrupole mass spectrometer (Pfeiffer PrismaPlus).

The FeO_*x*_/CeO_2_(111) samples were prepared in situ using two e-beam evaporators and examined by multiple methods without breaking the UHV. The chemical composition and thickness of the prepared FeO_*x*_/CeO_2_ layers for STM analysis were probed by UHV XPS. STM imaging was performed at 300 K using a combined STM/AFM Specs KolibriSensor.^22^ The pressure in the STM chamber during the UHV measurements was below 5 × 10^–10^ mbar. The microscope was operated in the constant current mode with current set points in the 6–15 pA range and sample voltages in the 2–4 V range. Nanonis electronics controlled the scanning process.

### 2.4 Computational Details

#### 2.4.1 Computational Methods

DFT calculations were carried out using the PW91 functional^23^ as implemented in the Vienna ab initio simulation package (VASP).^24−26^ The projector augmented wave method (PAW) was used to describe the interaction between fixed core electrons and explicitly described valence electrons. The PAW potentials describe 2s and 2p electrons explicitly for O, 3d and 4s electrons for Fe and 5s, 5p, 6s, 5d and 4f electrons for Ce. All structures were optimized with convergence criteria of 5 × 10^–6^ eV for total energies and 0.05 eV Å^–1^ for the forces acting on the atoms.

To properly describe electron localization on 3d states of Fe atoms and 4f states of Ce atoms, the GGA + U approach was employed to introduce energy penalties on partial occupations of Fe 3d and Ce 4f spin–orbitals. In line with previous studies, a U value of 5 eV was used for 3d states of Fe^27^ and 4 eV was used for 4f states of Ce.^28^ To describe electronic states involving different numbers of electrons transferred to the CeO_2_ support, we used the strategy described in our previous work.^100^

To account for van der Waals interactions when evaluating the stability of the different FeO_*x*_/CeO_2_(111) models considered, we have used the D2 correction of Grimme.^29,30^ Since there are no default parameters for the PW91+U approach used here (nor for plain PW91), we have used those parametrized for the similar PBE functional. We used *C*_6_ and *R*_0_ values of 0.7 J·nm^6^/mol and 1.342 Å for O,^29^ 10.8 J·nm^6^/mol and 1.562 Å for Fe,^29^ and 20.0 J·nm^6^/mol and 1.860 Å for Ce.^31,32^

#### 2.4.2 Structural Models

**Figure 1:**
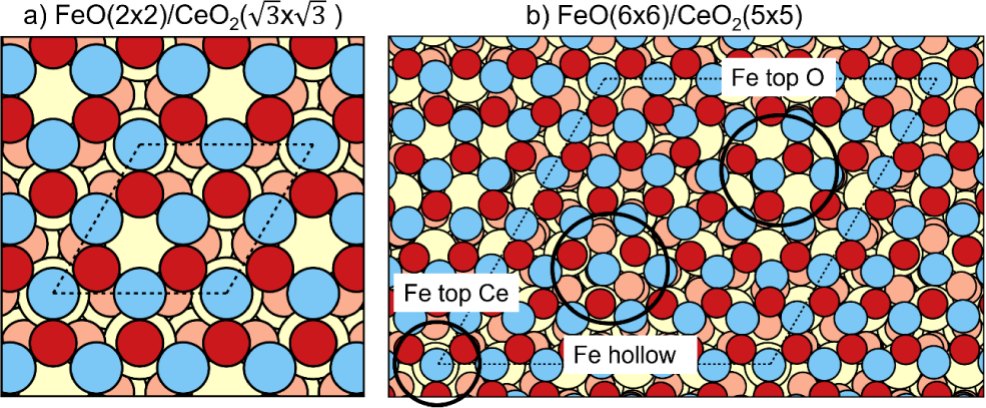
Structural models of the FeO/CeO_2_(111) system. FeO(2 × 2)/CeO_2_() (a) and FeO(6 × 6)/CeO_2_(5 × 5) (b) supercells. Blue, red, beige and orange circles correspond to Fe, O atoms in FeO and Ce, O atoms in CeO_2_, respectively. Dashed lines indicate the supercell boundaries. In (b), regions with different locations of Fe atoms with respect to the ceria surface are indicated.

Two different structural models were used to describe the interface between the 2D FeO thin film and the CeO_2_(111) support (Figure 1). The inherent mismatch between the 2D FeO and CeO_2_(111) lattices (with the PW91+U optimized lattice parameters of 3.35 and 5.48 Å, respectively) was solved by combining supercell lattices of FeO and CeO_2_(111) of different dimensions and adapting the lattice parameter of the ceria substrate to make commensurate supercell models of the interface. The first, smaller, model corresponds to a 2 × 2 supercell of the FeO monolayer over a  supercell of the CeO_2_(111) surface (Figure 1a). This results in a small compressive strain of the ceria surface of just 0.16%. Given the easily tractable size of the supercell (with 4 FeO units and 3 CeO_2_ surface units), this model is ideal for systematically evaluating different positions of the FeO ML with respect to the ceria surface (e.g., with a Fe atom located on top of either Ce, O or hollow sites of CeO_2_(111)) and different electronic states (i.e., with varying spin orientations and number of electrons transferred from FeO to the ceria substrate). The second, larger, model consists of a 6 × 6 supercell of FeO on a 5 × 5 supercell of CeO_2_(111) support (Figure 1b). Here, the ceria lattice parameter was stretched by 3.63%. This larger model is a better representation of the experimentally observed supercell and should better reproduce the coexistence of various Fe–substrate locations and the resulting long-range corrugation. In both models, 10.0 Å of vacuum were included in the *z* direction, and the CeO_2_(111) surface was described by 3 O–Ce–O trilayers, where only the bottom trilayer was kept fixed during structural relaxations.

## 3 Results

The study is structured into three distinct segments. Initially, we present two sets of SRPES experiments examining the incremental deposition of Fe onto the CeO_2_(111) surface at 300 K, as well as the stepwise annealing of the FeO_*x*_/CeO_2_(111) surface up to 700 K. These experiments provide valuable insights into the stability of the FeO_*x*_ layer and the intricate chemical interactions occurring between the FeO_*x*_ and CeO_2_ layers. Subsequently, we discuss the structure of the FeO_*x*_ layers and their chemical transformations characterized by in situ STM, LEED, and XPS. Finally, the results of the FeO_*x*_/CeO_2_(111) DFT calculations are discussed to delve deeper into the observed structures and their associated electronic structures and energetics.

### 3.1 Deposition of Fe on a Stoichiometric CeO_2_(111) Surface

**Figure 2:**
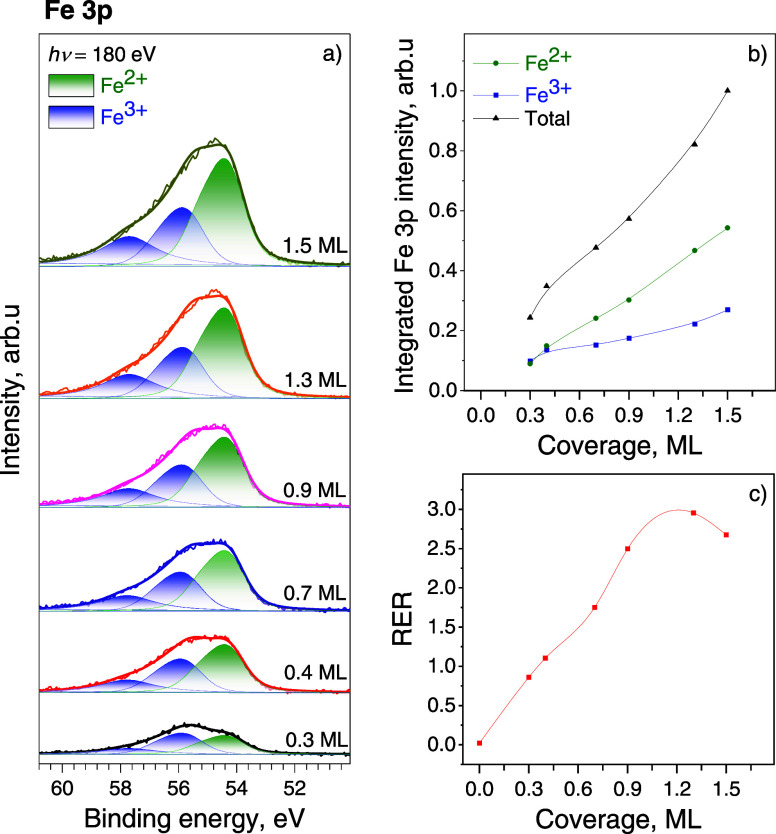
(a) Fe 3p SRPES spectra obtained during stepwise deposition of Fe onto the CeO_2_(111) surface at 300 K in UHV; (b) integrated intensities of Fe 3p spectral components; (c) the evolution of the RER shown as a function of Fe coverage.

Figure 2a presents Fe 3p SRPES spectra measured during stepwise Fe deposition on the CeO_2_(111) surface at 300 K in UHV (pressure ∼1 × 10^–9^ mbar). The obtained spectra were fitted with two unresolved doublets with a spin–orbital splitting of less than 1 eV. According to the literature, the doublet with the main peak position at about 55.7 eV and additional peak arising due to multiplet splitting at about 58 eV can be attributed to the formation of Fe^3+^ states.^33,34^ The doublet with the main peak at about 54.2 eV originates from the Fe^2+^ species.^33,34^ As can be seen from Figure 2b, both states gradually grew upon increasing the amount of Fe. In the case of 0.3–0.4 ML Fe/CeO_2_ surfaces, the concentrations of Fe^2+^ and Fe^3+^ were similar, whereas starting from the 0.4 ML coverage, Fe^2+^ states became dominant.

The corresponding Fe 2p core level spectra obtained by conventional XPS (Figure S1) also confirmed the formation of Fe^2+^ and Fe^3+^ cations. Since it is not easy to use the peak position of Fe 2p_3/2_ alone to distinguish between different oxidation states of Fe, they are usually detected from satellite features.^35^ In particular, FeO, consisting of Fe^2+^ ions, typically contains a prominent satellite feature at around ∼715 eV (6 eV above the main peak),^36^ while the broad satellite centered at ∼718 eV (8 eV above the main peak) is a characteristic of the Fe^3+^ state.^35^ During the stepwise deposition of Fe, the Fe 2p spectra revealed a minor satellite at approximately ∼718 eV and a progressively increasing peak at around 715 eV.^37^ These spectral features indicate a successive growth of Fe^2+^ and Fe^3+^ ion content on the CeO_2_ surface with each Fe deposition step. From the steeper growth of the Fe^2+^ peak, it can be concluded that Fe^3+^ is likely to be accommodated at the ceria interface while Fe^2+^ ions are formed in the upper layers. The formation of Fe^2+,3+^ states has been reported for Fe submonolayers on Al_2_O_3_,^38^ TiO_2_^39^ and ZnO^40^ supports, as well as for Fe single atoms on CeO_2_^41^ nanoparticles. These observations are also consistent with the general picture of the behavior of metals on oxide supports.^42^

Deposition of Fe onto the stoichiometric CeO_2_(111) surface at 300 K caused an immediate reduction of Ce^4+^ ions. The facile conversion of Ce^4+^ into Ce^3+^ is evident from the RER displayed in Figure 2c (for the corresponding VB spectra see Figure S2). Before the deposition of Fe, the RER was approximately 0.02, indicating the presence of a negligible amount of Ce^3+^ cations due to intrinsic defects in the surface region of the well-ordered CeO_2_(111) film.^43^ At the lowest Fe coverage (0.3 ML), the Ce^3+^ state started to appear and grew slowly with each addition of iron. Up to 1 ML coverage, iron uniformly reduced the ceria surface, increasing the contribution of Ce^3+^ steeply up to the value of 2.95. After exceeding the Fe coverage of 1.3 ML, the RER started to decrease, indicating that the Ce^3+^ content has reached saturation. The observed reduction of Ce^4+^ on the ceria surface can be explained by the charge transfer from the iron clusters to the ceria substrate^15^ and the migration of O atoms from the ceria surface to the supported Fe clusters. The charge transfer and concomitant reduction of Ce^4+^ to Ce^3+^ occurs via the formation of Fe–O bonds, created at the interface between the FeO_*x*_ clusters and the ceria surface and as a result of the O migration (reverse spillover) from the ceria surface to the FeO_*x*_ clusters.^41^ This reverse spillover, which involves the formation of O vacancies and has been observed for other ceria-supported metal particles,^44^ is the only mechanism that can explain the oxidation of noninterface Fe^2+^ and Fe^3+^ cations, which would repel each other in the absence of Fe–O–Fe motifs. Once all the surface oxygen is covered with a layer of FeO_*x*_ or the ceria surface is saturated with O vacancies, further charge transfer is hindered.

**Figure 3:**
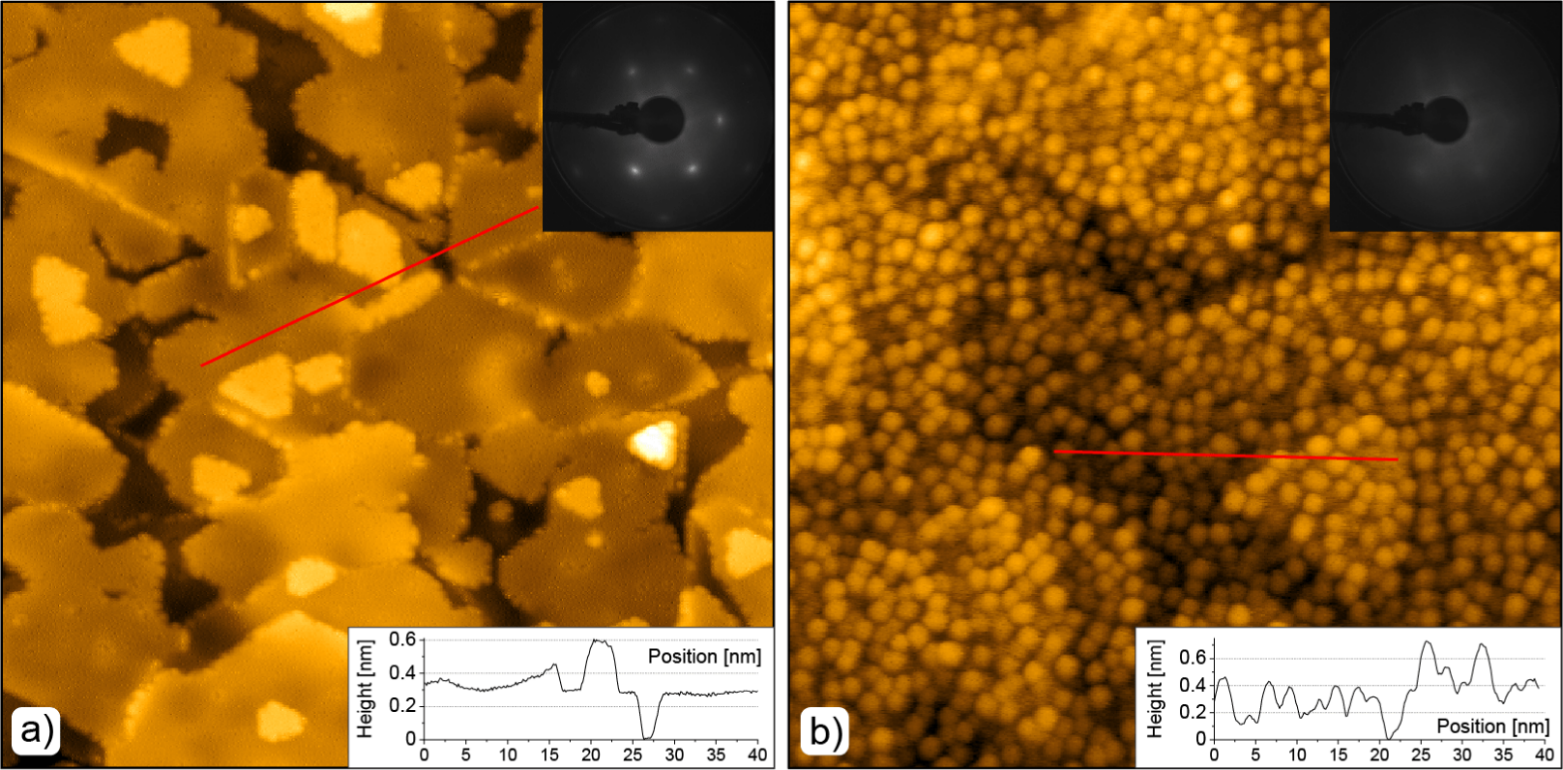
STM images of the clean CeO_2_(111) surface (a) and as-deposited 0.7 ML Fe/CeO_2_ surface (b). Parameters of the STM images: 80 × 80 nm^2^, *U*_s_ = 3.5 V, *I*_t_ ≈ 10 pA. LEED patterns of the CeO_2_(111) surface without/with Fe taken with a beam energy of 60 eV are shown in the top right insets. Red lines mark the positions of the height profiles shown in the bottom right insets.

To observe the surface morphology, a 0.7 ML Fe/CeO_2_(111) surface was characterized by STM. Figure 3a,b shows STM images obtained from the clean CeO_2_(111) and Fe/CeO_2_(111) surfaces, respectively. The thin film of CeO_2_(111) exhibited a well-ordered structure with a continuous layer terminated by atomically flat terraces, in agreement with our previous studies.^21^ The LEED pattern obtained from this film confirmed its crystallinity. After about 0.7 ML of Fe was deposited on the surface at 300 K in UHV, small circular islands appeared (Figure 3b). These islands were from about 0.2 nm up to 0.6 nm in height, up to 3 nm in diameter and were uniformly distributed across the surface. LEED observation shows almost complete disappearance of the diffraction spots, indicating that the ceria surface was covered by a disordered layer of clusters. These circular entities were interpreted as nonstoichiometric FeO_*x*_ clusters by PES. The formation and random distribution of the FeO_*x*_ clusters indicates that Fe does not preferentially agglomerate at ceria steps. This is further supported by our experiments with a lower 0.2 ML coverage of FeO_*x*_ (see Figure S6). This behavior can be attributed to the high oxophilicity of iron atoms.^45,46^ Due to their high affinity for oxygen and presumably low mobility at 300 K, Fe atoms most likely tend to localize on O sites on the stoichiometric CeO_2_(111) terraces, where they experience high diffusion barriers.^46^ Therefore, their growth is dominated by the formation of irregular nanoparticles over the entire surface of the substrate rather than the preferential nucleation on the surface defects.

### 3.2 Oriented FeO Layer Formation on CeO_2_(111)

**Figure 4:**
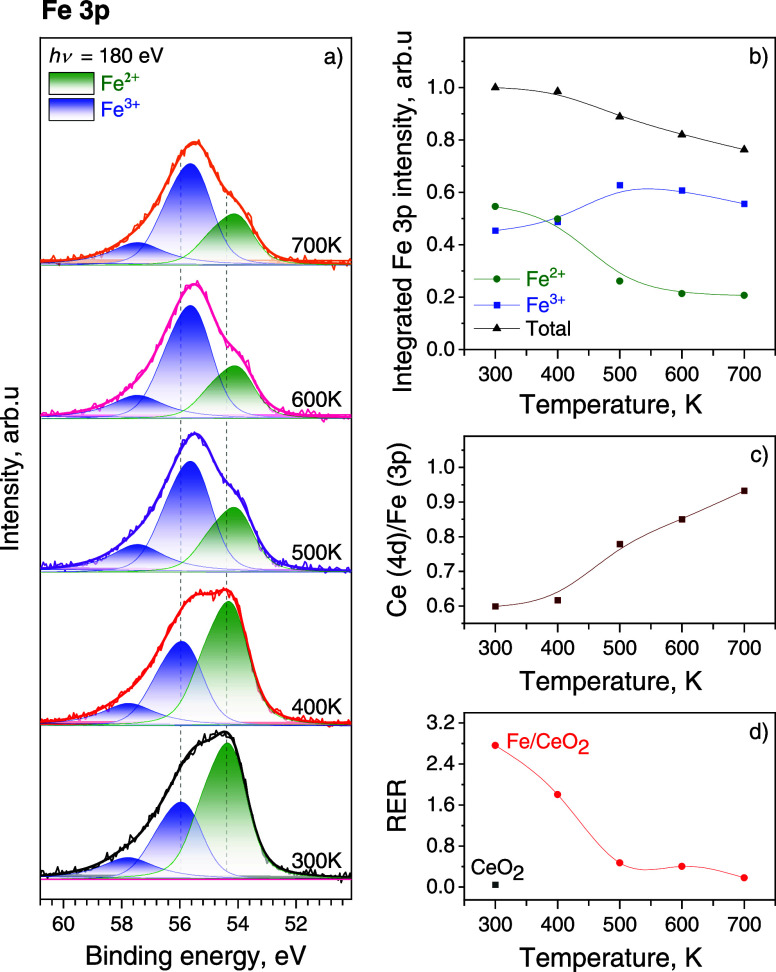
(a) Fe 3p SRPES spectra acquired from the 0.7 ML of Fe on the CeO_2_(111)/Cu(111) system in UHV at different temperatures; (b) integrated intensities of the Fe 3p spectral components, (c) Ce/Fe surface atomic ratio and (d) RER as a function of temperature.

**Figure 5:**
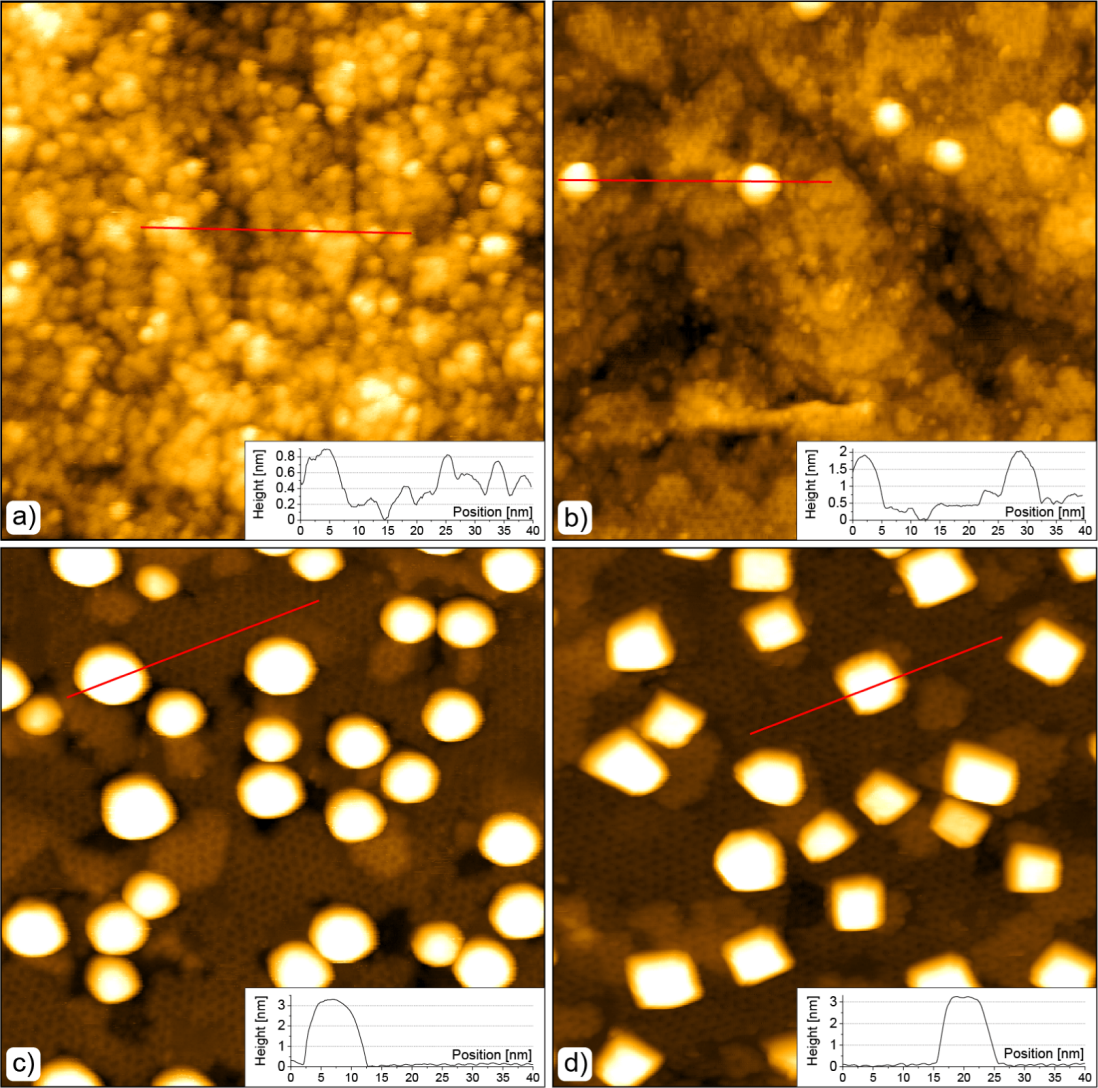
STM images of the CeO_2_(111) surface upon deposition of Fe and subsequent annealing in UHV and oxygen. Top: ∼1 ML of Fe was deposited on the CeO_2_(111) surface at 300 K and annealed at 400 K (a), and 600 K (b) in UHV. Bottom: 2 ML of Fe was deposited on the CeO_2_(111) surface at 600 K in 1 × 10^–8^ mbar of O_2_ (c) and annealed at 600 K for 20 min in 1 × 10^–8^ mbar of O_2_ (d). All images have the size of 80 × 80 nm^2^ and were obtained with *U*_bias_ ≈ 3 V and *I*_t_ ≈ 10 pA. Red lines mark positions of the height profiles shown in bottom right insets.

In the next step, the 0.7 ML Fe/CeO_2_(111) system was stepwisely annealed in UHV (pressure ∼1 × 10^–9^ mbar) up to 700 K. The evolution of the Fe 3p spectra is shown in Figure 4a. We noted that the total spectral intensity within the Fe 3p region remained relatively stable up to 400 K. However, a gradual decrease becomes evident as temperature increases above this point (Figure 4b). An analysis of the Ce/Fe ratio, derived from the Ce 4d and Fe 3p spectral areas divided by corresponding photoionization cross sections as a function of annealing temperature, also revealed a significant decrease in the intensity of the iron signal compared to cerium. These observations strongly suggest changes in the morphology of the deposited iron. As shown in STM images with a higher surface coverage (1–2 ML) in Figure 5, this is partially due to the formation of large FeO_*x*_ particles on the surface. Specifically, the growth of these larger FeO_*x*_ clusters is expected to expose a greater portion of the substrate, thereby contributing to the increase in the Ce/Fe ratio, as shown in Figure 4c. In addition to the surface changes of the FeO_*x*_ layer, this ratio can also be increased by thermally induced diffusion of Fe ions into the bulk of ceria.^12^ Furthermore, the elevated temperature promoted the oxidation of Fe ions to a higher valence state. The calculated relative intensities of Fe^2+^ and Fe^3+^ components presented in Figure 4b show that the dominant Fe^2+^ contribution began to drop already at 400 K, and upon reaching a temperature of 700 K, its intensity decreased more than 2-fold in favor of the Fe^3+^ state.

Simultaneously with the oxidation of Fe, a gradual decrease in the amount of Ce^3+^ ions was observed (Figures 4d and S3). The fact that the spectral signal for Fe^3+^ and Ce^4+^ states increased during UHV annealing can be explained by the oxygen diffusion from deeper ceria layers to the surface, typically observed within the temperature range of 470–600 K.^47^ This diffusion process together with direct adsorption of oxygen from a residual atmosphere led to the replenishment of surface oxygen vacancies, which had initially formed due to the Fe–CeO_2_ interaction, and to further oxidation of iron.^48^ The accumulation of additional oxygen on the surface, leading to the change in the surface potential, can also explain an observed slight shift of the Fe 3p spectral components to a lower binding energy.

The STM images obtained after annealing of ∼1 ML of Fe on the CeO_2_(111) surface at 400 and 600 K in UHV (pressure ∼1 × 10^–9^ mbar) are shown in Figure 5a,b, respectively. Upon annealing at 400 K, the size and distribution of FeO_*x*_ clusters did not change significantly from the ones observed in Figure 3b. However, at 600 K, STM images revealed the appearance of significantly larger FeO_*x*_ particles up to 1.5 nm in height and up to 8 nm in apparent lateral size. Additionally, a hexagonal structure covering the rest of the ceria surface emerged. Based on its orientation and visible parameters of the lattice, the structure was interpreted as a moiré pattern, which in STM images arises as a result of electronic or morphological interference between two crystal lattices with the same symmetry but different periodicity.^49,50^ In this case, we observe the overlap of an oriented 2D FeO_*x*_ layer and the CeO_2_(111) substrate. To the best of our knowledge, the growth of the thin FeO_*x*_ layer on the CeO_2_(111) substrate has not been reported to date. Nonetheless, the amount of oriented iron oxide is relatively low when the Fe/CeO_2_(111) system undergoes annealing in UHV. This is attributed to a substantial portion of iron being incorporated into large amorphous particles, resulting in the above-mentioned lowering of the Fe 3p SRPES signal.

To enhance the formation of the 2D oriented iron oxide overlayer and simultaneously investigate its correlation with the presence of FeO_*x*_ particles, we deposited 2 ML of Fe onto the CeO_2_(111) substrate at 600 K in 1 × 10^–8^ mbar of O_2_. The resulting surface topography can be seen in Figure 5c. Under these conditions, the formation of round FeO_*x*_ clusters with heights up to 4 nm and apparent lateral sizes up to 20 nm was observed. The rest of the ceria surface was uniformly covered by the 2D FeO_*x*_ layer, the arrangement of which was substantially improved by elevated oxygen pressure. The deposited layer contains a relatively low number of defects, concentrated mostly in the vicinity of CeO_2_ and FeO_*x*_ step edges. After prolonged annealing in O_2_ (Figure 5d), the clusters transition from round hemispherical shapes to faceted pyramidal structures, indicating a structural shift from amorphous to crystalline. In contrast to the striking oxidation-induced morphological changes of the large FeO_*x*_ clusters, the 2D layer remains unchanged. This indicates that the 2D FeO_*x*_ layer is formed relatively quickly after the deposition at 600 K, while remaining Fe atoms can diffuse more freely on the FeO_*x*_-passivated surface and agglomerate into large clusters.

**Figure 6:**
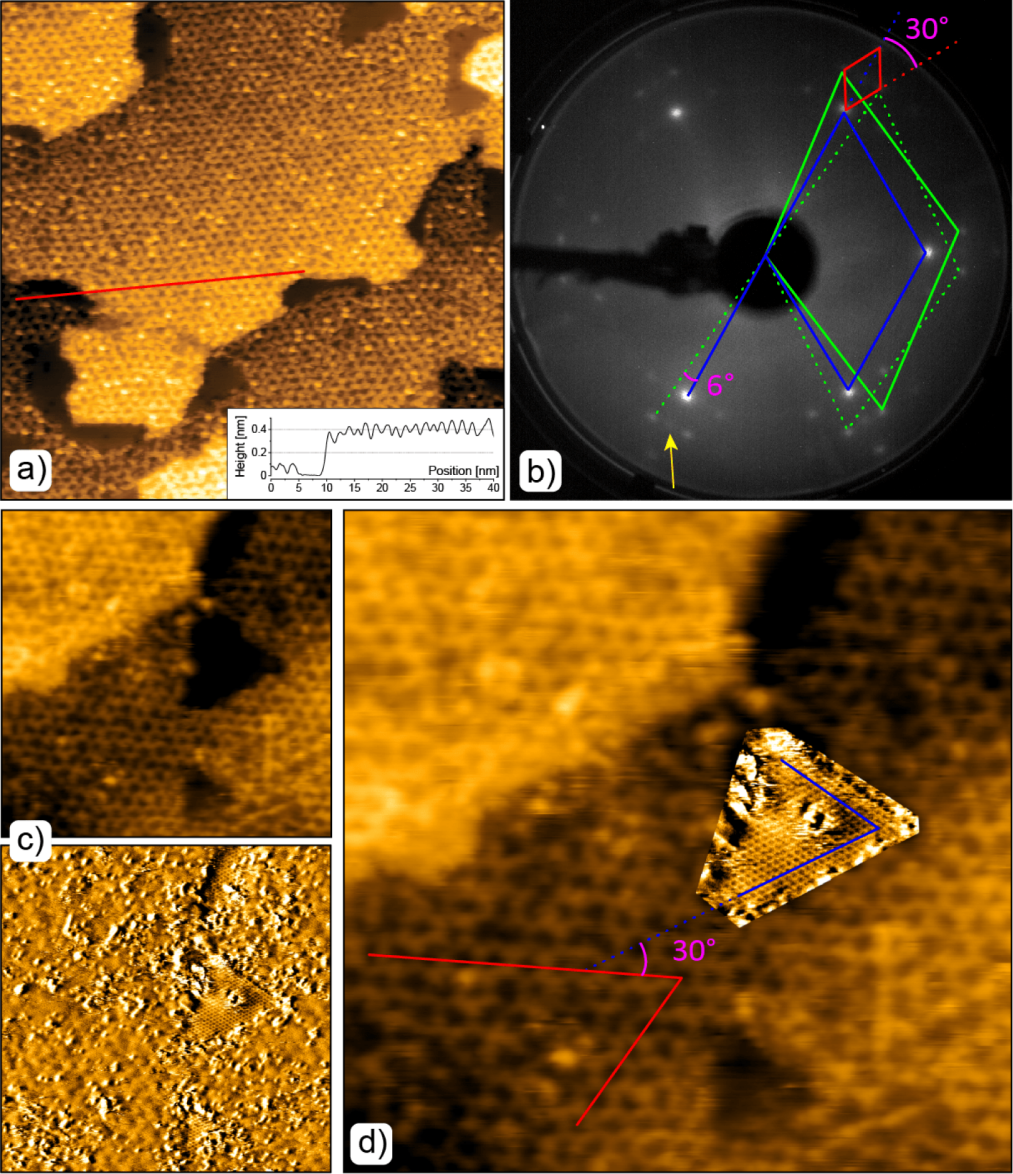
Structure of the 2D FeO_*x*_ layer. (a) STM image of the moiré superstructure on the FeO_*x*_/CeO_2_(111) surface after annealing at 700 K in 1 × 10^–8^ mbar of O_2_. Surface area 70 × 70 nm^2^, *U*_bias_ ≈ 3 V, *I*_t_ ≈ 10 pA. (b) LEED image obtained from the same surface. Electron energy: 50 eV. The CeO_2_(111), FeO_*x*_ and the moiré superstructure elementary cells are marked by blue, green and red parallelograms, respectively. A diffraction spot corresponding to a minority surface structure with the FeO_*x*_ structure aligned with the main direction of CeO_2_(111) is marked by a yellow arrow. (c) Two simultaneously captured STM images showing the structure of the CeO_2_ lattice and the moiré superstructure on the FeO_*x*_ layer. The top image represents the height channel of the constant current STM image, covering a surface area of 30 × 30 nm^2^, with *U*_bias_ set at 1.6 V and *I*_t_ ≈ 10 pA. The bottom image displays the frequency shift channel as measured by a Kolibri sensor.^22^ (d) A composite image comprising parts of (c) illustrating the relative orientation of the two structures. Blue and red lines mark the main directions of the ceria and the superstructure lattices, respectively. Relative angles between the structures are marked in (b) and (d).

To further study the structure of the 2D FeO_*x*_ layer, unaffected by the amorphous FeO_*x*_ clusters, we deposited 0.7 ML of Fe onto the CeO_2_ at 300 K in UHV and annealed the sample at 700 K in 1 × 10^–8^ mbar of O_2_. This procedure resulted in the surface being completely covered by the moiré superstructure with a relatively high degree of order (Figure 6a), enabling us to determine the structure of the FeO_*x*_ layer. Specifically, having calibrated the STM data by the uncovered CeO_2_ patches (Figure 6c,d), we determined that the elementary moiré cell is rotated by approximately 30° with respect to the CeO_2_ lattice and has a periodicity almost four times greater than the CeO_2_ lattice. Based on the lattice constants and the relative orientation of the FeO_*x*_ layer, it can be determined that the FeO_*x*_ unit cell is rotated by ±6° with respect to the CeO_2_ unit cell and that the lattice constant of the FeO_*x*_ layer is  = (0.31 ± 0.01) nm. The atomic periodicity of 0.31 nm corresponds to those obtained for FeO/Pt(111) (0.311 nm)^49^ and FeO/Ru(0001) (0.308 nm)^51^ and is only slightly higher than that expected from the theoretical calculations of FeO.^52^

The validity of the measured FeO lattice parameters was also corroborated by the LEED analysis (Figure 6b). Bright, hexagonally arranged spots, marked by a blue parallelogram, correspond to the CeO_2_(111)–(1 × 1) structure.^21^ Satellite diffraction peaks observed in the vicinity of the main ceria diffraction spots arose due to the superposition of two metal-oxide lattices and multiple elastic scattering of diffracting electrons on both gratings,^53^ resembling the superstructures reported for FeO(111)/Pt(111) and FeO(111)/Au(111) surfaces.^49,54^ By comparing the distances of the diffraction spots from the zeroth series with the distances of the (1 × 1) spots of the CeO_2_ substrate, we obtained the lattice constants of the deposited layer (see green parallelograms) and the moiré superstructure (see a red parallelogram) to be  = (0.31 ± 0.01) nm and *a*_moiré_ = (1.55 ± 0.04) nm, respectively. Furthermore, the angle between the moiré superstructure and the CeO_2_ lattice was determined to be (30 ± 1)°, corresponding to the (6 ± 1)° angle between the main direction of the (1 × 1) structure and the FeO structure.^53^ Both of these values agree very well with the values measured from the STM images further indicating that the 2D FeO-like phase is indeed formed on the surface of CeO_2_. Additionally, the presented LEED image features an extra set of spots marked by a yellow arrow. These spots are located in the main crystallographic directions of the CeO_2_(111)–(1 × 1) structure but correspond to the lattice constant of about 0.31 nm. We assign these spots to a minority FeO-like structure which is aligned with the ceria substrate. The substrate-aligned minority superstructures were also observed in our STM images (see Figure S4).

**Figure 7:**
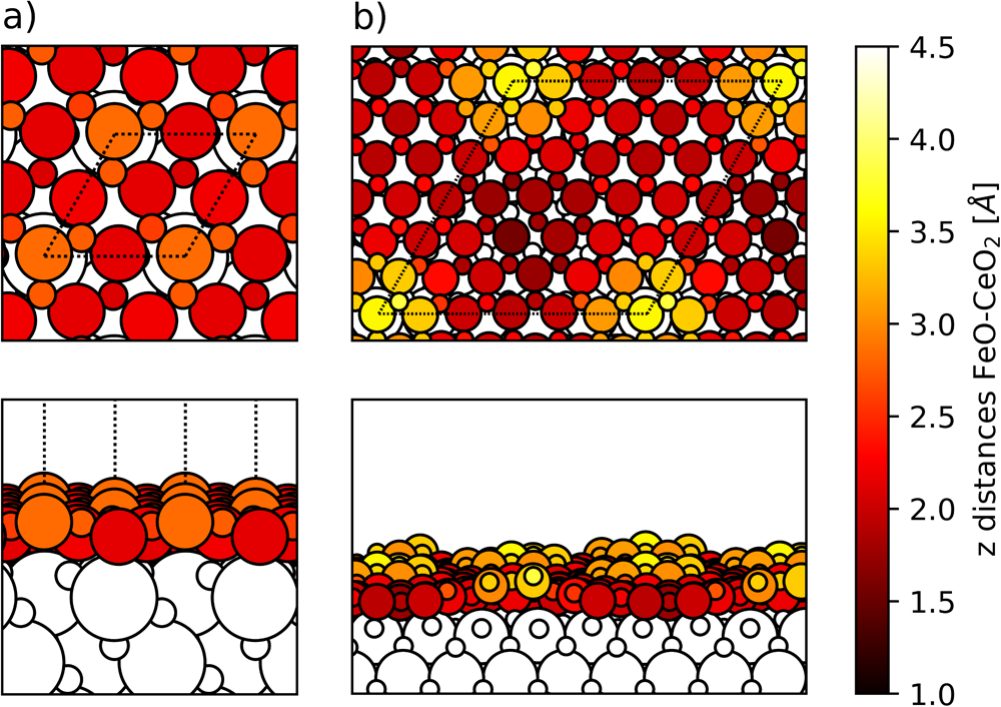
Height profile of the FeO monolayer supported on CeO_2_ for the (a) FeO(2 × 2)/CeO_2_(√3 × √3) and (b) FeO(6 × 6)/CeO_2_(5 × 5) models. Upper and lower panels correspond to top and side views, respectively. The Fe (big circles) and O (small circles) atoms of the FeO monolayer are colored depending on their distance to the CeO_2_ support according to the color bar. Distances are calculated with respect to the outermost O atoms of the bare CeO_2_(111) surface. The white circles indicate Ce (big circles) and O (small circles) atoms of the CeO_2_ surface.

The structural properties of the FeO/CeO_2_(111) interface were further elucidated by means of the DFT+U calculations described in the computational details section. For the smaller FeO(2 × 2)/CeO_2_(√3 × √3) structural model (Figure 1a), we have evaluated three different orientations of the FeO ML corresponding to one of the 4 Fe atoms of the FeO supercell placed on top of an O atom, a Ce atom, or a hollow site of the ceria substrate, respectively. We note that these alternative arrangements involve different interactions between the O_FeO_ and the ceria surface, which also affects the overall stability of each structure. The most stable structure found for this model is illustrated in Figures 1a and 7a, exhibiting both an Fe atom and an O_FeO_ atom on top of two different Ce atoms of the ceria surface. The different interactions of Fe and O atoms with the ceria surface lead to a corrugation of ∼0.7 Å, with the Fe and O_FeO_ atoms found at a 3.51 and 2.84 Å distance above the underlying Ce atoms, respectively. The calculated adhesion energy *E*_adh_ between the FeO monolayer and the ceria surface is −0.47 eV per FeO unit (calculated as *E*_adh_ = *E*(FeO/CeO_2_) – *E*(FeO) – *E*(CeO_2_)). This energy gain is larger than the −0.36 eV energy difference between 2D FeO and the most stable (monoclinic/halite) phase of bulk FeO, which indicates that formation of the FeO/CeO_2_ heterostructure is more thermodynamically favorable than agglomerated 3D particles of FeO. A large fraction (−0.20 eV) of this adhesion energy (−0.47 eV) corresponds to van der Waals interactions.

The optimized structure of the larger FeO(6 × 6)/CeO_2_(5 × 5) model is illustrated in Figure 7b and exhibits a more substantial corrugation of ∼2.3 Å. The lowest-lying region of the FeO ML corresponds to that with Fe atoms bonding to O atoms of the ceria surface, with Fe– distances of ∼1.9–2.2 Å (similar to the 1.93 Å Fe–O distances in the free-standing FeO ML). These bonds are formed for several Fe atoms near top positions of  atoms and for one Fe atom on the hollow site between 3  atoms. In the middle-lying region, the O_FeO_ atoms neighboring the Fe atom are also close to Ce atoms of the ceria surface, forming O_FeO_–Ce bond distances between 2.6 to 2.7 Å, close to those of the Ce–O bonds in the clean ceria surface (2.43 Å). These Fe– and O_FeO_–Ce distances reflect a strong interaction between the FeO ML and the ceria surface. Indeed, for the FeO(6 × 6)/CeO_2_(5 × 5) model, *E*_adh_ is of −0.45 eV per FeO unit, further confirming the high stability of the FeO/CeO_2_ interface. The highest-lying regions of the FeO ML correspond, in turn, to Fe atoms on top of Ce atoms of the ceria surface, with Fe–Ce distances of 4.34 Å. Thus, an apparent repulsion between Fe and Ce cations emerges from both FeO(6 × 6)/CeO_2_(5 × 5) and FeO(2 × 2)/CeO_2_(√3 × √3) models, although the larger supercell can accommodate more pronounced corrugations leading to significantly longer Fe–Ce distances.

We also compare the stability of the FeO/CeO_2_(111) system to that of Fe-doped CeO_2_(111), which has been proposed as a relevant component for catalysts based on Fe and CeO_2_.^4,6^ To do so, we compare the adhesion energy of FeO to CeO_2_(111) to the energy of the Fe-doping reaction using the same 2D FeO reference and a 2 × 2 supercell of the CeO_2_(√3 × √3) surface: FeO + (*x*/2)O_2_ Ce_36_O_72_ → FeCe_35_O_71+*x*_ + CeO_2_, where FeCe_35_O_71+*x*_ corresponds to the Fe-doped CeO_2_(2√3 × 2√3) surface (see Figure S7) and x takes values of 1, 0, or −1 for the Fe-doped surface when fully oxidized, with one O vacancy, or with two O vacancies, respectively. FeO corresponds to the 2D FeO monolayer, CeO_2_ corresponds to bulk ceria, and Ce_36_O_72_ is the slab model of the CeO_2_(2√3 × 2√3) supercell. *E*_dop_ is thus calculated as *E*_dop_ = *E*(FeCe_35_O_71+*x*_) + *E*(CeO_2_) – *E*(FeO) – *E*(Ce_36_O_72_) – (*x*/2)*E*(O_2_). The calculated *E*_dop_ values are 0.66, 1.09, and 2.66 eV per Fe atom, for the Fe-doped CeO_2_(111) system with zero, one, and two vacancies, respectively. Considering the calculated *E*_adh_ (−0.47 eV), the ceria supported FeO monolayer is at least 1.13 eV more stable than Fe-doped CeO_2_(111), which explains why Fe dissolution into the ceria lattice is very limited.

**Figure 8:**
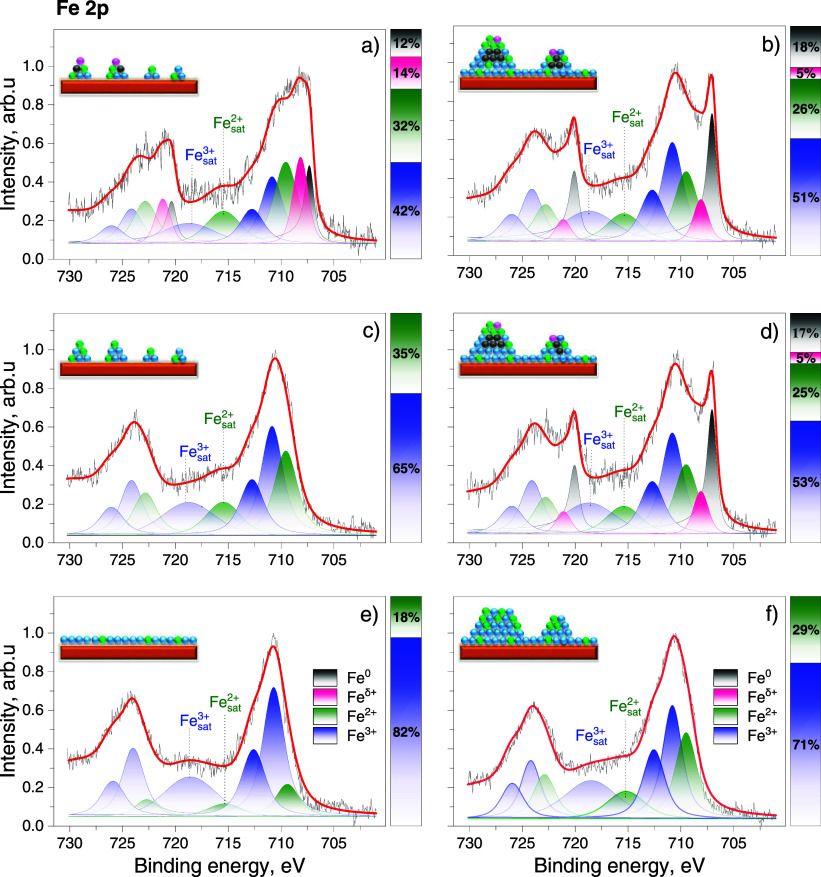
In situ XPS Fe 2p core level spectra showing the evolution of FeO_*x*_/CeO_2_ (111) samples with 0.7 ML Fe coverage (left) and 2 ML Fe coverage (right) after Fe deposition (a, b), STM measurement (c, d) and annealing in 1 × 10 ^–8^ mbar of O_2_ (e, f). The spectra were acquired with Al Kα radiation (1486.6 eV) and normalized to the same maximum height. Relative intensities of Fe^3+^, Fe^2+^, Fe^δ+^ (δ ϵ (0, 2)) and Fe^0^ spectral components determined from the peak areas are specified in the columns.

The STM and LEED investigation was also accompanied by in situ XPS measurements aiming to study the surface chemistry of the resulting FeO_*x*_/CeO_2_ systems. Two notable sets of Fe 2p spectra are presented in Figure 8 and corresponding Ce 3d spectra in Figure S5. The top-left spectrum in Figure 8 corresponds to the surface covered by relatively small FeO_*x*_ clusters with a high surface-to-volume ratio, as depicted in Figure 3b. Upon deposition of Fe in UHV, this system initially contained the metallic Fe^0^ and other oxidized states of iron. We note that Fe^δ+^ probably corresponds to just partially oxidized Fe atoms, which have donated electrons to the ceria surface but without transforming into an oxidized phase of Fe. Similar δ+ atomic charges have been detected for other ceria-supported transition metals.^55,56^ It is worth noting that we observed a gradual decrease of Fe^0^ and Fe^δ+^ and a simultaneous increase of Fe^2+^ and Fe^3+^ components during the spectrum acquisition in Figure 8a (acquisition time was around 1 h). Subsequently, upon finishing LEED and STM experiments (after 24 h), the Fe^0^ and Fe^δ+^ components disappeared completely, as shown in Figure 8c, while the FeO_*x*_ on the surface remained in the form of small clusters. The spontaneous, albeit slow, oxidation of Fe^0^ and Fe^δ+^ species in UHV is an indication of O migration from the ceria surface to the supported FeO_*x*_ particles. After annealing this surface at 700 K in 1 × 10^–8^ mbar of O_2_, the FeO_*x*_ rearranged and formed the well-ordered 2D FeO_*x*_ layer, as shown in Figure 6, with about a 5:1 Fe^3+^/Fe^2+^ ratio (Figure 8e).

The Fe 2p spectrum in Figure 8b corresponds to the surface featuring large FeO_*x*_ clusters, as illustrated in Figure 5c. Similar to the surface with small clusters, the XPS spectrum contains higher oxidation states of Fe as well as states attributed to Fe^δ+^ and Fe^0^, despite iron being deposited onto the surface in the 1 × 10^–8^ mbar of O_2_. Obviously, the oxygen supplied during the deposition at 600 K was not sufficient to fully oxidize the iron deposit, resulting in the presence of Fe^0^ species. This metallic iron is presumably located within noninterfacial regions (i.e., bulk and/or surface positions of the upper layers) of the large clusters. After the following STM and LEED measurements (Figure 8d), the average Fe oxidation state on the surface remained almost unchanged, indicating that the interface FeO_*x*_ region or the FeO_*x*_ layer on the surface of large clusters impedes further oxygen diffusion into the clusters at 300 K. Complete oxidation of Fe was only achieved after an additional 20 min annealing in 1 × 10^–8^ mbar of O_2_ at 600 K, as shown in the bottom-right spectra of Figure 8. At this stage, the Fe^3+^/Fe^2+^ ratio is about 2.5:1, indicating that even after prolonged oxygen exposure at elevated temperatures, Fe atoms within FeO_*x*_ clusters are, on average, less oxidized than those within the 2D FeO_*x*_ layer.

In the SRPES experiment presented in Figure 4 we gradually annealed the sample in UHV. The Fe^3+^/Fe^2+^ ratio measured from the Fe 3p spectra stabilized at about 3:1, aligning more closely with the scenario observed with large FeO_*x*_ clusters, as determined by XPS. The Fe^0^ component in this set of spectra was not observed from the beginning, indicating rapid oxidation of rather small clusters immediately after deposition. Similar to the surface depicted in Figure 5a,b, the small clusters annealed under relatively low oxygen pressure may have coalesced into larger clusters, leaving the CeO_2_ surface only partially covered by the 2D FeO_*x*_ layer. The coalescence of small FeO_*x*_ particles is further evidenced by the gradual decrease in the total spectral intensity of the Fe 3p peak (Figure 4b). Furthermore, disparities in the SRPES and XPS spectra are attributable to the higher surface sensitivity of SRPES (180 eV vs 1487 eV energy of the primary radiation results in information depths of 2 vs 5 nm, respectively). Consequently, the SPRES spectra are more indicative of the Fe^3+^/Fe^2+^ ratio in the topmost surface layer of the FeO_*x*_ clusters, whereas XPS provides a representation of the ratio across entire clusters.

**Figure 9:**
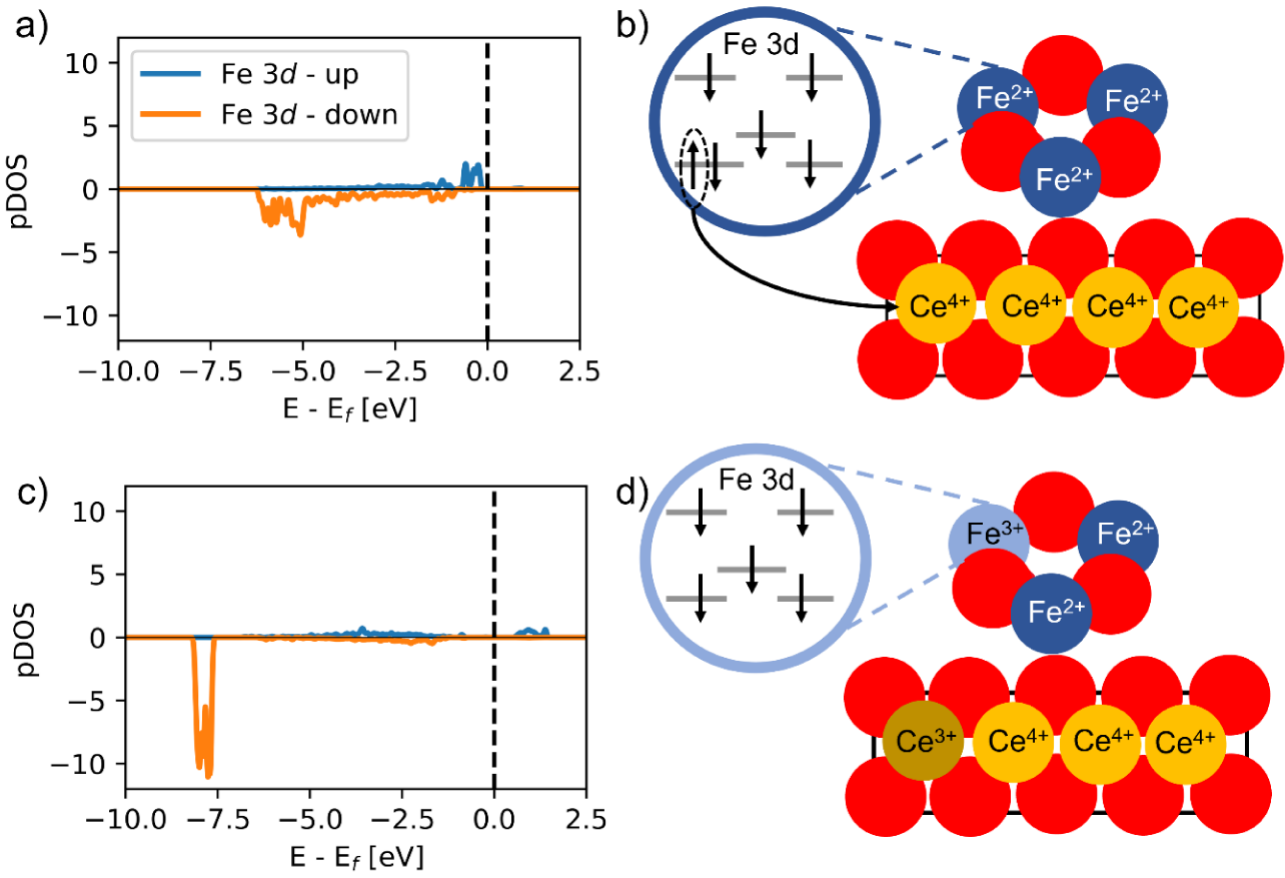
Electronic structure of Fe atoms in the FeO/CeO_2_(111) system for different electronic states without (a and b) or with (c and d) transferred electrons from Fe 3d to Ce 4f orbitals. The density of states projected in the 3d states of Fe are shown in (a) and (c), and schemes illustrating formal Fe and Ce charges and the electronic configuration of the Fe 3d states are shown in (b) and (d).

A dominant Fe^2+^ oxidation state of Fe atoms in the 2D layer is expected based on its structural parameters corresponding to the FeO phase, however, a dominant Fe^3+^ state (up to 5:1 ratio) is observed from our spectroscopy experiments. We address this discrepancy by means of DFT calculations. The Bader atomic charges, projected density of states (pDOS), and magnetic moments derived from the DFT calculations for the different models allow us to further characterize the electronic structure of the FeO/CeO_2_(111) interface. We have focused on evaluating the charge distribution and comparing the stability between electronic states differing in the spin-alignment of the Fe cations and on the number of transferred electrons from the FeO ML to the underlying ceria substrate. As illustrated in Figure 9, this redox process leads to the oxidation of one Fe^2+^ cation to Fe^3+^ and the concomitant reduction of one Ce^4+^ cation to Ce^3+^. The formal charges of the Fe and Ce cations can be inferred from their Bader charges and magnetic moments. Upon oxidizing Fe^2+^ to Fe^3+^, the (positive) Bader charge increases from 1.4 |e| to 1.8 |e| and the absolute magnetic moment increases from ∼3.7 to ∼4.1 μ_B_. This indicates that the donated electron is that with the opposite spin orientation than the Fe cation, consistently with Hund’s rule and as illustrated in the Fe 3d pDOS in Figure 9a,c. In turn, Ce^3+^ centers exhibit a lower (positive) Bader charge than Ce^4+^ and a characteristic magnetic moment close to ∼0.9 μ_B_ due to the occupation of a Ce 4f spin–orbital.

**Figure 10:**
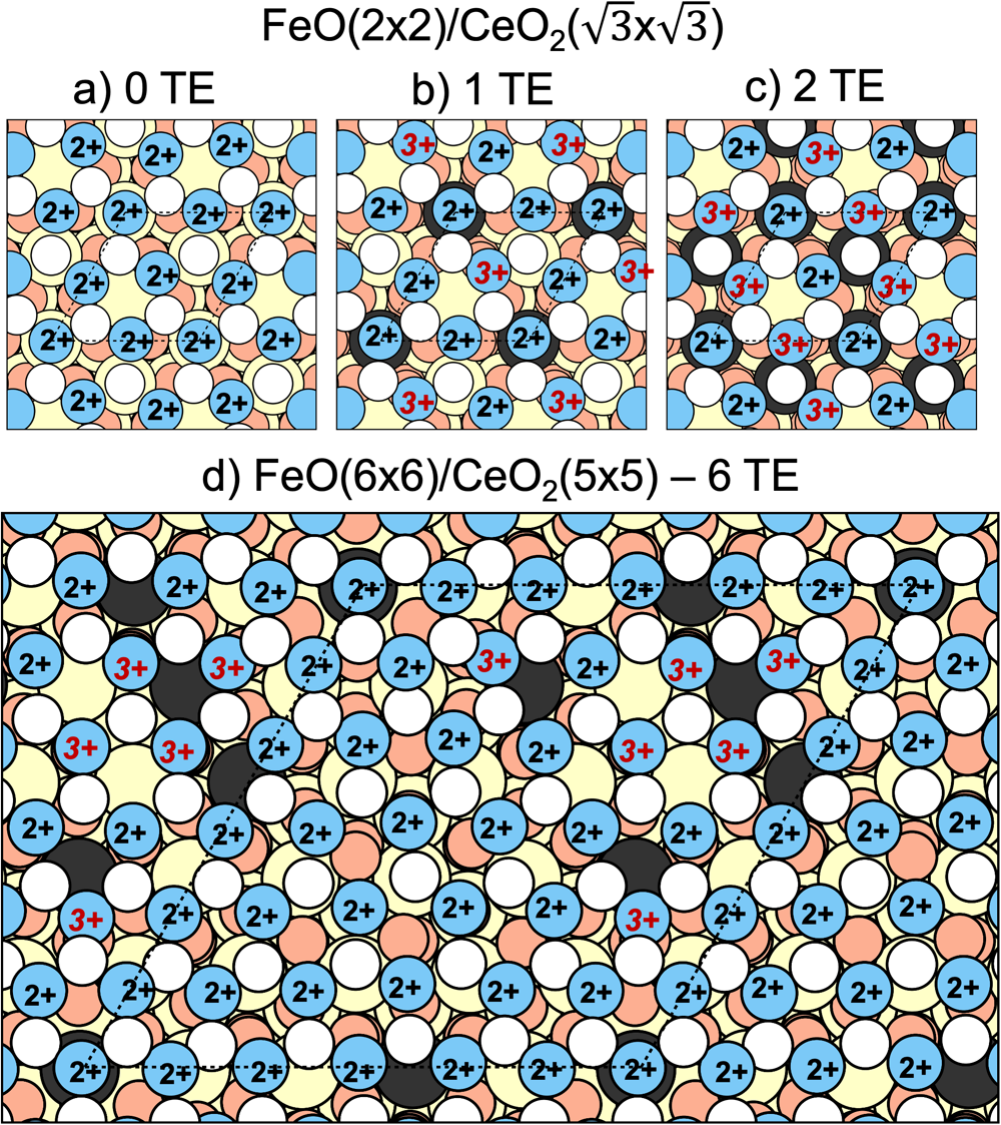
Different electronic states differing in the number of transferred electrons (TE) from FeO to CeO_2_ in the FeO(2 × 2)/CeO_2_() (a, b, and c) and FeO(5 × 5)/CeO_2_(6 × 6) (d) models. Blue, white, beige, black and orange circles correspond to Fe, O_FeO_, Ce^4+^, Ce^3+^ and O_ceria_ atoms, respectively. The labels above Fe atoms indicate their oxidation state (2+ or 3+).

For the most stable structure of the smaller model, we have identified three different electronic states with zero, one, or two electrons transferred from FeO to CeO_2_(111), represented in Figure 10a–c. We note that as for the free-standing FeO monolayer, the most stable electronic states for the FeO/CeO_2_(111) system exhibit an antiferromagnetic ordering of the Fe cations, independently of them being Fe^2+^ or Fe^3+^. The most stable electronic state found for the FeO(2 × 2)/CeO_2_(√3 × √3) model contains only one Fe^3+^ cation (one transferred electron from FeO to CeO_2_). The states with two or zero transferred electrons are 0.56 and 0.40 eV (0.14 and 0.1 eV per FeO unit) less stable, respectively. The most stable state with one transferred electron corresponds to a 1:3 Fe^3+^/Fe^2+^ ratio and 1/3 of outermost Ce atoms reduced to Ce^3+^. The concentration of Fe^3+^ is therefore lower than that measured for the FeO on CeO_2_ system annealed at high temperatures.

For the larger FeO(6 × 6)/CeO_2_(5 × 5) model, we have only sampled a single electronic state due to the large computational cost of every structural relaxation. This single optimization has converged to an electronic state with 6 Fe^3+^ cations, corresponding to a 1:5 ratio between Fe^3+^ and Fe^2+^ cations, where 6 of the 25 outermost Ce cations are reduced to Ce^3+^. The distribution of the Fe^2+^, Fe^3+^, Ce^3+^, and Ce^4+^ cations is illustrated in Figure 10d. We have not exhaustively sampled all possible electronic states differing in the number and position of the Ce^3+^ cations formed, but for the larger model, Ce^3+^ centers generally form in the vicinity of Fe^3+^ centers.

**Figure 11:**
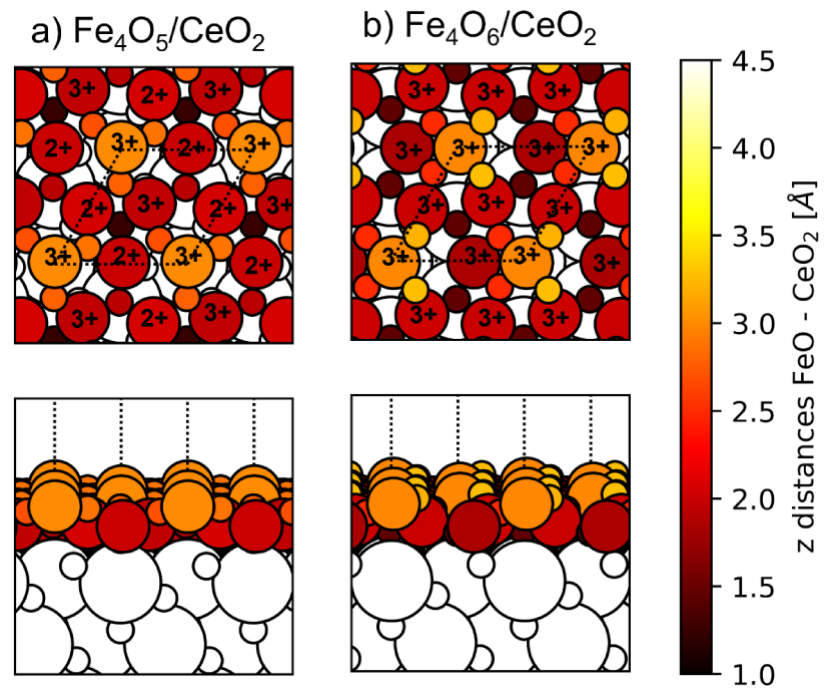
Oxidation of the FeO(2 × 2)/CeO_2_() model. Structure and Fe oxidation state for the adsorption of one (a) and two (b) O atoms on FeO(2 × 2)/CeO_2_(), leading to Fe_4_O_5_ and Fe_4_O_6_ stoichiometries, respectively, of the FeO_*x*_ monolayer. White circles indicate Ce (big circle) and O (small circle) atoms of the CeO_2_ support, while Fe (big colored circle) and O (small colored circle) are colored to indicate their vertical distance to the bare CeO_2_ support according the color bar on the right-hand side.

The distribution of Fe oxidation states for both FeO/CeO_2_(111) models indicates that the FeO monolayer is only partially oxidized by the ceria surface in the absence of O_2_. To evaluate how exposure to O_2_ would affect this distribution, we have carried out calculations with one or two additional O atoms on the FeO(2 × 2)/CeO_2_() model (see Figure 11). The most stable adsorption sites for O on this system correspond to hollow sites between three Fe cations and right on top of a Ce atom of the ceria surface. O atoms adsorbed on these positions bond to the 3 Fe cations and to the Ce below, with Ce–O distances of 2.3 Å. The system with one adsorbed O atom (Figure 11a) has 2 Fe^3+^ and 2 Fe^2+^ cations, indicating that this adsorption reoxidized one Ce^3+^ to Ce^4+^ and one Fe^2+^ to Fe^3+^, therefore also leading to the oxidation of the Ce^3+^ center that had formed upon interaction with FeO back to Ce^4+^. Upon adsorption of the second O atom (Figure 11b), the two remaining Fe^2+^ cations are oxidized to Fe^3+^. The adsorption energy *E*_ads_(O) (calculated as *E*_ads_(O) = *E*[O-surface] – *E*[surface] – 0.5*E*[O_2_]) of the first and second O atoms is −0.72 and −1.26 eV, respectively, which indicates that the oxidation of FeO (Fe_4_O_4_) to Fe_2_O_3_ (Fe_4_O_6_) is thermodynamically favorable. Here, we just added O atoms in three-Fe-coordinated sites and relaxed the structure. A more exhaustive search for stable structures of the Fe_2_O_3_ monolayer is likely to result in even more favorable oxidation of the ceria-supported 2D FeO.

The activity toward oxidation reactions of catalysts based on Fe and CeO_2_ has been linked to the effect of Fe dopants on the oxygen storage capacity of ceria.^5,6^ Since the oxygen storage capacity of ceria-based systems depends on their reducibility, their formation energy *E*_f_(O_vac_) is often used as a descriptor for activity.^57,58^ Therefore, to establish differences between Fe-doped CeO_2_(111) and the FeO_*x*_/CeO_2_(111) systems, we have calculated *E*_f_(O_vac_) for their corresponding models (Figures S7 and S8). *E*_f_(O_vac_) is calculated as *E*_f_(O_vac_) = *E*(X–O_vac_) – *E*(X) – 0.5*E*(O_2_), where *E*(X–O_vac_) and *E*(X) are the energies of the considered systems with and without the formed vacancy, respectively.

We start from the most oxidized states (i.e., Fe_4_O_6_/Ce_9_O_18_ for FeO_*x*_/CeO_2_(111) and FeCe_35_O_72_ for Fe-doped CeO_2_(111)). For FeO_*x*_/CeO_2_(111), the first, second, and third O vacancies have *E*_f_(O_vac_) values of 1.26, 0.72, and 2.99 eV, respectively. For Fe-doped CeO_2_(111), the first and second O vacancies result in *E*_f_(O_vac_) of 0.43 and 1.56 eV, respectively. These results indicate that it is not significantly more favorable to form vacancies in Fe-doped CeO_2_(111) than on the 2D FeO_*x*_/CeO_2_(111) system, and that both systems have a rich redox chemistry with readily available (i.e., low *E*_f_(O_vac_)) O atoms.

## 4 Discussion

As mentioned above, PES and STM results indicate that nonstoichiometric FeO_*x*_ clusters are formed as a result of iron–ceria interaction. The process can be described by the reaction *y*Fe + *x*CeO_2_ → *x*CeO_(2–*y*)_ + *y*FeO_*x*_, where *x* = 0···1.5 and *y* = 0···0.5. Interestingly, the molar enthalpy change (Δ*H*_f^298^_) for the full redox reaction Fe + 2CeO_2_ → Ce_2_O_3_ + FeO involving bulk oxides is 109.2 kJ/mol,^59^ suggesting that the process is thermodynamically unfavorable at 300 K. However, it is thermodynamically more favorable to partially reduce the CeO_2_(111) surface than bulk ceria,^58^ and to partially oxidize Fe particles than bulk Fe. A similar phenomenon has also been reported for other systems, such as Ni/CeO_2_ or Co/CeO_2_, where the formation of CoO^60^ and NiO^61^ clusters was observed. The oxidation of supported metal particles by lattice O atoms of CeO_2_(111) is typically enabled by reverse spillover mechanism, which has been reported for even more noble metals such as Pt.^44^ The spontaneous oxidation of ceria-supported 3D Fe particles in UHV thus confirms that a reserve spillover mechanism is enabled in the FeO_*x*_/CeO_2_(111) interface.

We have shown that Fe atoms, which form small clusters upon deposition, are readily oxidized to varying degrees. Conversely, bigger FeO_*x*_ clusters only oxidize after prolonged annealing in O_2_ at 600 K. The slower oxidation kinetics observed in the bulk or upper layers of large FeO_*x*_ clusters, even at elevated temperatures, suggests relatively lower oxygen mobility within the superficial or interfacial iron oxide, which likely encapsulates and passivates metallic cores of the Fe clusters.

In a 2D configuration, the (111) planes of both CeO_2_ ( = 0.382 nm)^62^ and FeO ( = 0.31 nm)^49^ surfaces exhibit hexagonal symmetry, indicating their potential for epitaxial arrangement. Although significant lattice mismatches, such as that between the FeO and CeO_2_(111) (exceeding 20%), often prevent epitaxial growth, that is not always the case. Instead of just the lattice mismatch, the growth of one oxide on another is also governed by the stability of the oxide–oxide interface and the process kinetics.^63^ The modification of the surface free energy Δγ induced by a film formation on a substrate can be written as Δγ = γ_f_ + γ_i_ – γ_s_, where γ_f_ and γ_s_ are surface free energies of a film and a substrate, respectively, while γ_i_ is the interface energy. If Δγ < 0, the formation of a wetting layer is energetically favored, while Δγ > 0 would lead to islanding or clustering. Taking γ_f_ as that of O-terminated FeO(111) (1.3 J/m^2^),^64^ γ_s_ as that of O-terminated CeO_2_(111) (0.7 J/m^2^),^65^ and the γ_i_ of −0.7 J/m^2^ (−0.47 eV/FeO unit) calculated by DFT in this work for the FeO/CeO_2_(111) interface, results in Δγ = −0.1 J/m^2^, indicating a preference for FeO film formation on ceria. This is in line with the slight preference to form the FeO/CeO_2_(111) interface rather than bulk FeO derived solely from our DFT calculations. In addition to being thermodynamically favorable, the formation of the FeO films on CeO_2_(111) may be dominated also by other factors such as the high degree of reduction of the ceria surface after the deposition of Fe or the different formation and oxidation kinetics of 2D films and 3D FeO_*x*_ nanostructures.

Nevertheless, our DFT calculations showed that the interaction of the 2D FeO_*x*_ layer with ceria is strengthened by the structural corrugation of the 2D FeO_*x*_ layer (which can be observed experimentally by STM), by van der Waals interactions, by the further oxidation of the 2D FeO_*x*_ layer upon incorporation of additional O atoms, or by the transfer of electrons from the 2D FeO layer to the ceria substrate. The latter is analogous to the electronic metal–support interactions widely reported for ceria-supported metals.^56,66^

## 5 Conclusions

This model catalyst study offers valuable insights into the intricate behavior of iron in real Fe/CeO_2_ catalysts.^3,4,6,7^ The presented findings shed light on the growth dynamics and interaction between iron and ceria during thermal treatment under UHV conditions and in the presence of O_2_. Our data demonstrate that iron readily undergoes oxidation upon deposition, giving rise to small FeO_*x*_ clusters on the ceria substrate at 300 K. Upon annealing in UHV, some clusters grow into larger particles, while others disperse and form a thin FeO_*x*_ layer. Annealing in an oxygen-rich environment promotes the dispersion of iron on the ceria surface and enhances the ordering of the resulting 2D FeO_*x*_ thin film, as validated by STM and LEED. By means of DFT calculations, we showed that this 2D surface layer is thermodynamically more stable than bulk FeO or Fe-doped CeO_2_(111) due to (i) the formation of bonds between the Fe atoms and the O atoms of the ceria lattice, (ii) van der Waals interactions between the FeO_*x*_ layer and the CeO_2_(111) surface, (iii) the structural corrugation of the FeO monolayer, (iv) the transfer of electrons from Fe to ceria, and (v) the additional oxidation by adsorbed oxygen.

In addition, we have proposed a method for producing stable, well-ordered epitaxial FeO_*x*_ films on a ceria support. Similar ultrathin FeO films grown on much more expensive noble metal substrates have demonstrated exceptional catalytic activity in CO oxidation reactions.^67^ Thanks to the structural corrugation of the 2D FeO_*x*_ layer, this system could also serve as an excellent template for supporting ordered arrays of noble-metal nanoparticles.^68,69^ Lastly, the capacity of the 2D FeO film to donate electrons suggests that the 2D FeO/CeO_2_(111) system can be used as a reducing support for catalytically active metal particles, enabling the synthesis of catalytic materials with negatively charged metal centers.

## Supporting Information Available

Additional Fe 2p spectra fitting parameters, Fe 2p core-level spectra, valence band spectra, STM images, in situ Ce 3d core-level spectra, and DFT supporting calculations (PDF)
