# Supplementary material for: Electronic and Structural Properties of Thin Iron Oxide Films on CeO2
Source: ACS Appl Mater Interfaces. 2024 Aug 21;16(35):46858–71. doi: 10.1021/acsami.4c05542 (PMC11378155; doi:10.1021/acsami.4c05542)
Supplement: Supplementary file 1 — am4c05542_si_001.pdf [file am4c05542_si_001.pdf]

Supporting Information

Electronic and Structural Properties of Thin Iron Oxide Films on CeO_2

Lesia Piliai[1,#], Pablo Castro-Latorre[2,#], František Pchálek[1], Shiva Oveysipoor[1], Yuliia Kosto[1,3], Ivan Khalakhan[1], Tomáš Skála[1], Konstantin M. Neyman[2,4], Pere Alemany[2], Michael Vorochta[1], Albert Bruix[2,], Peter Matvija[1,*], Iva Matolínová[1]*

[1] Department of Surface and Plasma Science, Faculty of Mathematics and Physics, Charles University, V Holešovičkách 2, 180 00 Prague 8, Czech Republic

[2] Departament de Ciència de Materials i Química Física and Institut de Química Teòrica i Computacional (IQTCUB), Universitat de Barcelona, 08028 Barcelona, Spain

[3] Brandenburg University of Technology Cottbus-Senftenberg, Applied Physics and Semiconductor Spectroscopy, Konrad-Zuse-Strasse 1, 03046 Cottbus, Germany

[4] ICREA (Institució Catalana de Recerca i Estudis Avançats), 08010 Barcelona, Spain

[#] These authors contributed equally to the work and are considered co-first authors.

[*] Corresponding authors:
Peter Matvija: peter.matvija@mff.cuni.cz
Albert Bruix: abruix@ub.edu

Keywords: ceria, CeO_2, iron oxide, 2D layer, catalysis, STM, XPS, DFT

The Fe 2p band exhibits a complex shape characterized by peak asymmetries, d–d multiplet splitting, and shake-up satellites typical of transition metals and their oxides. In particular, the multiplet splitting in Fe 2p spectra is due to unpaired electrons resulting in several final states in the band. The Fe^{2+} cations in the $3d^6$ initial state have no unpaired electrons, resulting in a low spin state (S = 0). Therefore, no multiplet splitting is expected for low spin Fe^{2+} cations. In contrast, the Fe^{3+} cations have unpaired electrons in the $3d^5$ initial state, resulting in a high spin state (S = 5/2)[1]. Consequently, the Fe^{3+} state undergoes multiplet splitting due to the coupling of the final states with the unpaired 3d electrons, resulting in a broadened spectrum. Studies on iron oxides such as Fe_2O_3, Fe_3O_4, and FeOOH have demonstrated that the broadening of Fe 2p spectra comprises not only one asymmetric peak but the summation of several symmetric peaks.[2–6] In our study, we adapted a fitting procedure proposed by Pauly et al. utilizing the smallest number of peaks necessary to represent the experimental data accurately.[4] Specifically, two Voigt doublets were employed to fit the Fe^{3+} state, often referred to Fe^{3+} species with tetrahedral and octahedral occupancies.[7–11] One Voigt doublet was utilized for the fitting of Fe^{2+} and $Fe^{\delta+}$ oxidation states, while the Doniach–Šunjić doublet was employed to represent the Fe^0 state. We believe that $Fe^{\delta+}$ corresponds to partially oxidized FeO_x nanoparticles. Satellite features were fitted with the single Voigt peak. The fitting parameters are summarized in Table S1.

Table S1. The results of XPS Fe 2p spectra fitting for metallic Fe, Fe_2O, FeO and Fe_2O_3

	Fe^0	$Fe^{\delta+}$	Fe^{2+}	$Fe^{3+}_{(Oct)}$	$Fe^{3+}_{(Tet)}$
Binding energy Fe $2p_{3/2}$	707.2[12,13]	708.1[14]	709.5[7,15]	710.8[7,13,16]	712.7[7,16]
Spin-orbit splitting (eV)	13[17]	13	13.3	13.3[8]	13.3[8]
Branching ratio	0.5	0.5	0.52	0.52[8]	0.52[8]
Lorentzian width (eV)	0.6	0.6	1.6	1.6	1.7
Gaussian width (eV)	0.6	1.4	1.6	1.4	1.6
Binding energy satellite (eV)	-		715.4[15]	718.7[15]	

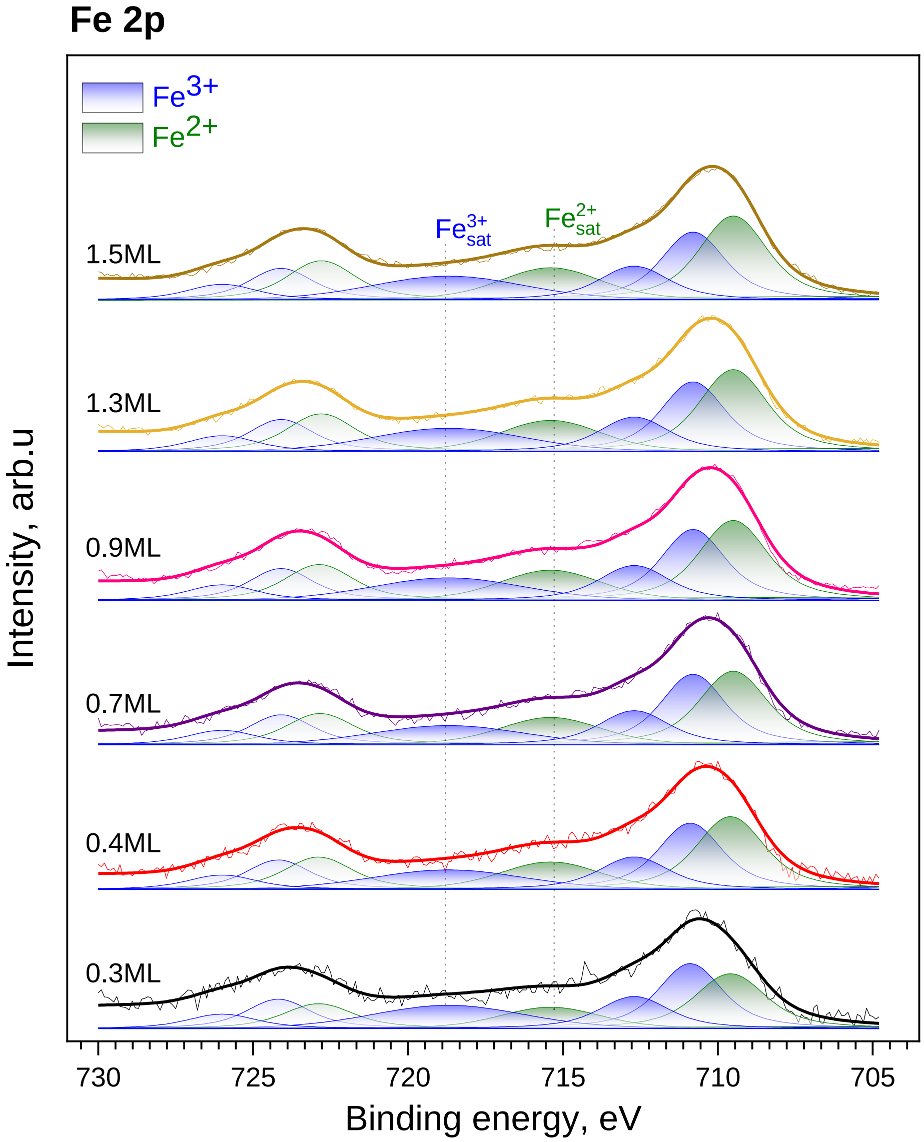

Figure S1. Fe 2p core level spectra obtained by XPS for stepwise deposition of iron on ceria. The spectra were acquired with Mg Kα radiation (1253.6 eV) and normalized to maximum height.

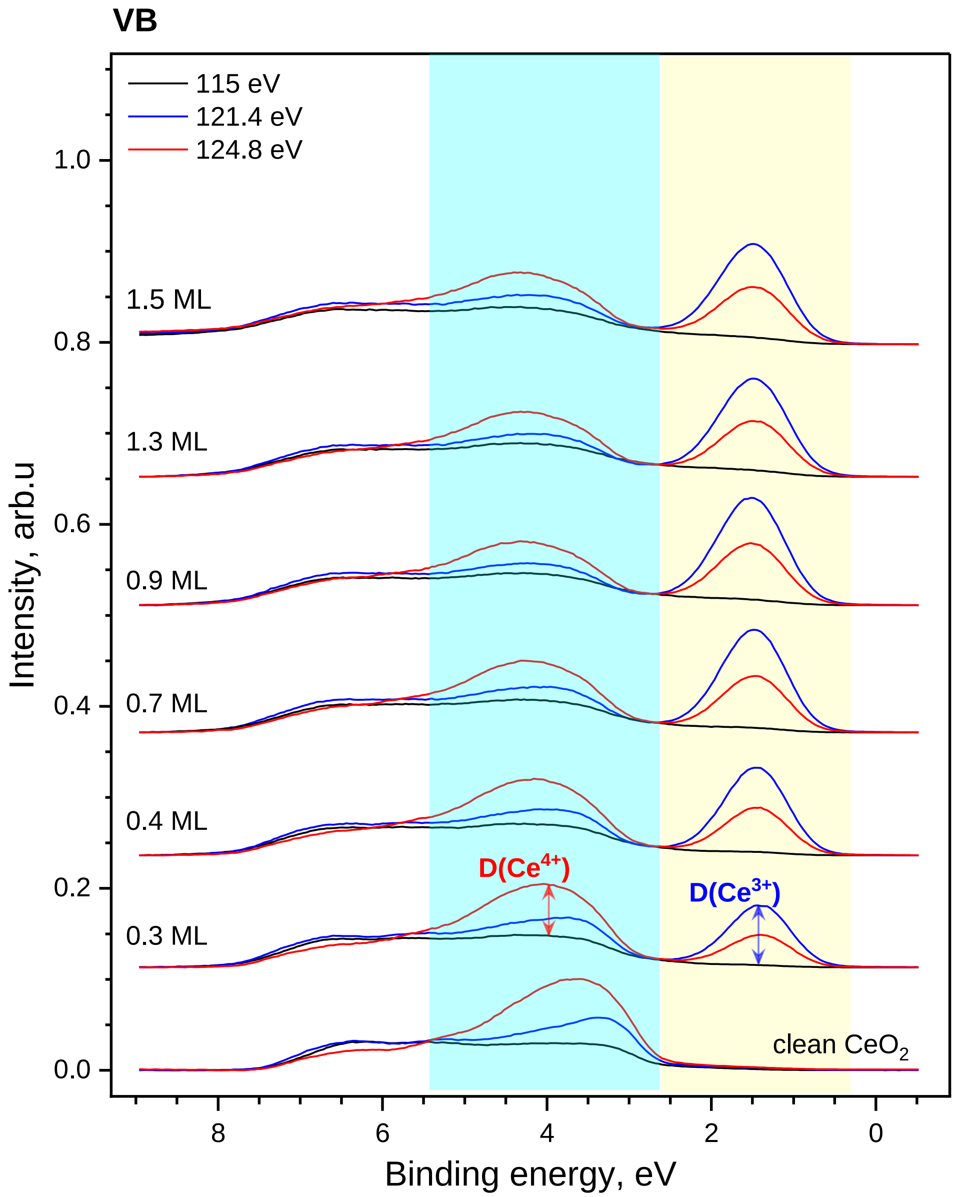

Figure S2. Valence band (VB) spectra obtained during stepwise deposition of Fe onto the CeO_2(111) surface at 300 K in UHV at 115 eV (off-resonance), 121.4 eV (Ce^{3+} resonance) and 124.8 eV (Ce^{4+} resonance) photon energy. The arrows show resonance enhancements D(Ce^{3+}) and D(Ce^{4+}).

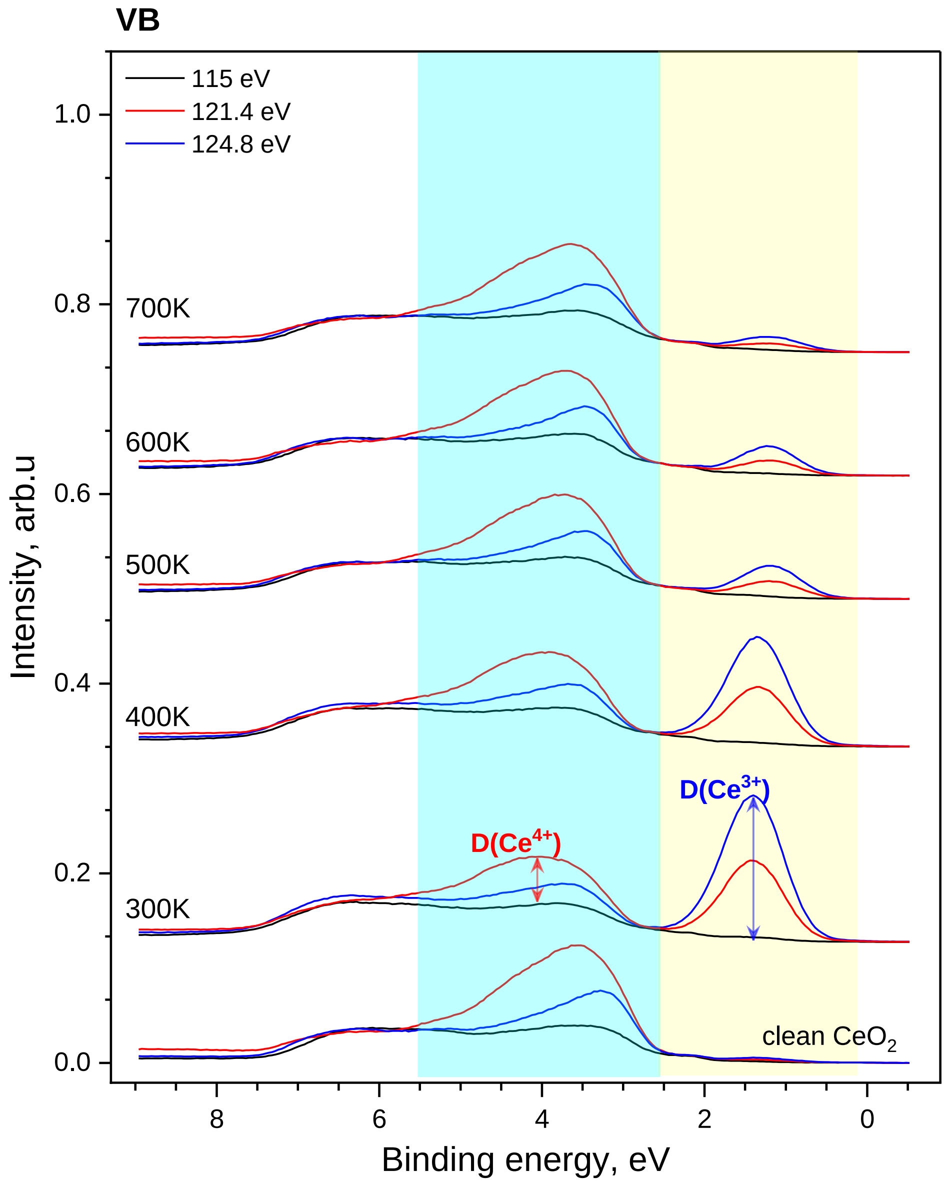

Figure S3. Valence band (VB) spectra acquired from the 0.7 ML of Fe on the CeO_2(111)/Cu(111) system in UHV at different temperatures at 115 eV (off-resonance), 121.4 eV (Ce^{3+} resonance) and 124.8 eV (Ce^{4+} resonance) photon energy. The arrows show resonance enhancements D(Ce^{3+}) and D(Ce^{4+}).

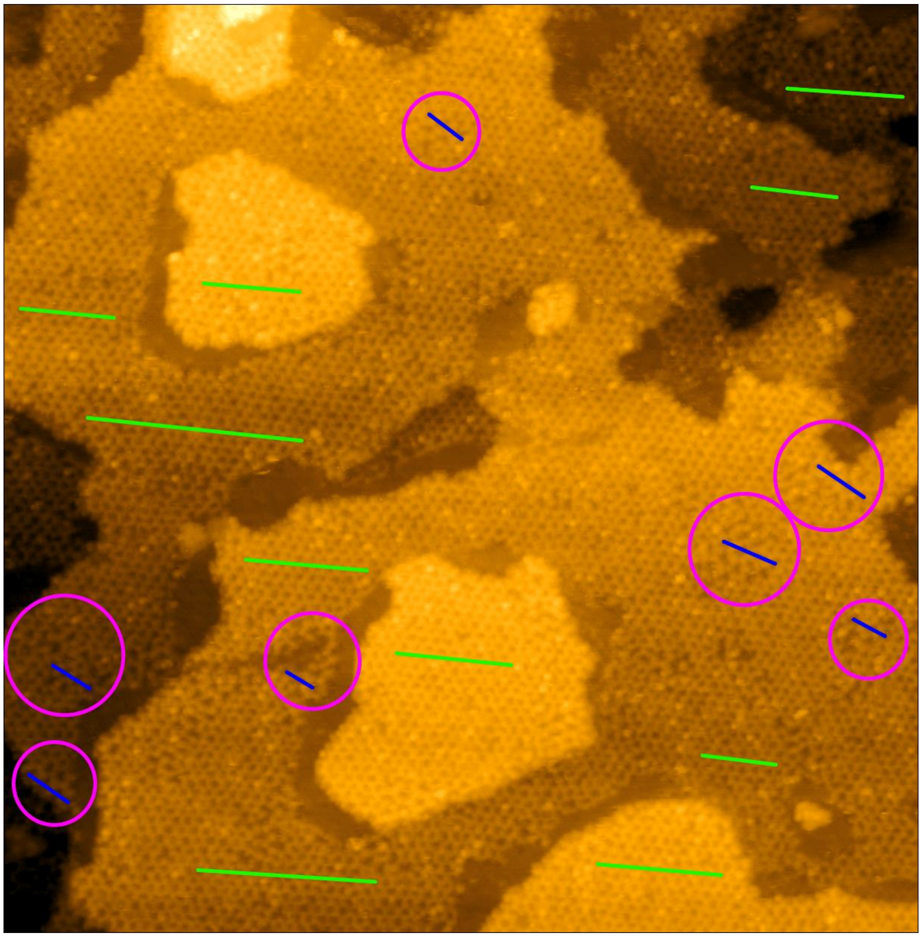

Figure S4. STM image of the $FeO_x/CeO_2(111)$ surface prepared by deposition of about 0.7 ML of Fe on the $CeO_2(111)$ substrate at 300 K and annealing at 800 K in 1×10^{-8} mbar of O_2. Surface area 110×110 nm^2, $U_{bias} \approx 3$ V, $I_t \approx 10$ pA. Most of the surface is covered by the FeO_x superstructure (marked by green lines) rotated by 30° with respect to the substrate lattice. Minority superstructures better aligned with the substrate are marked by magenta circles.

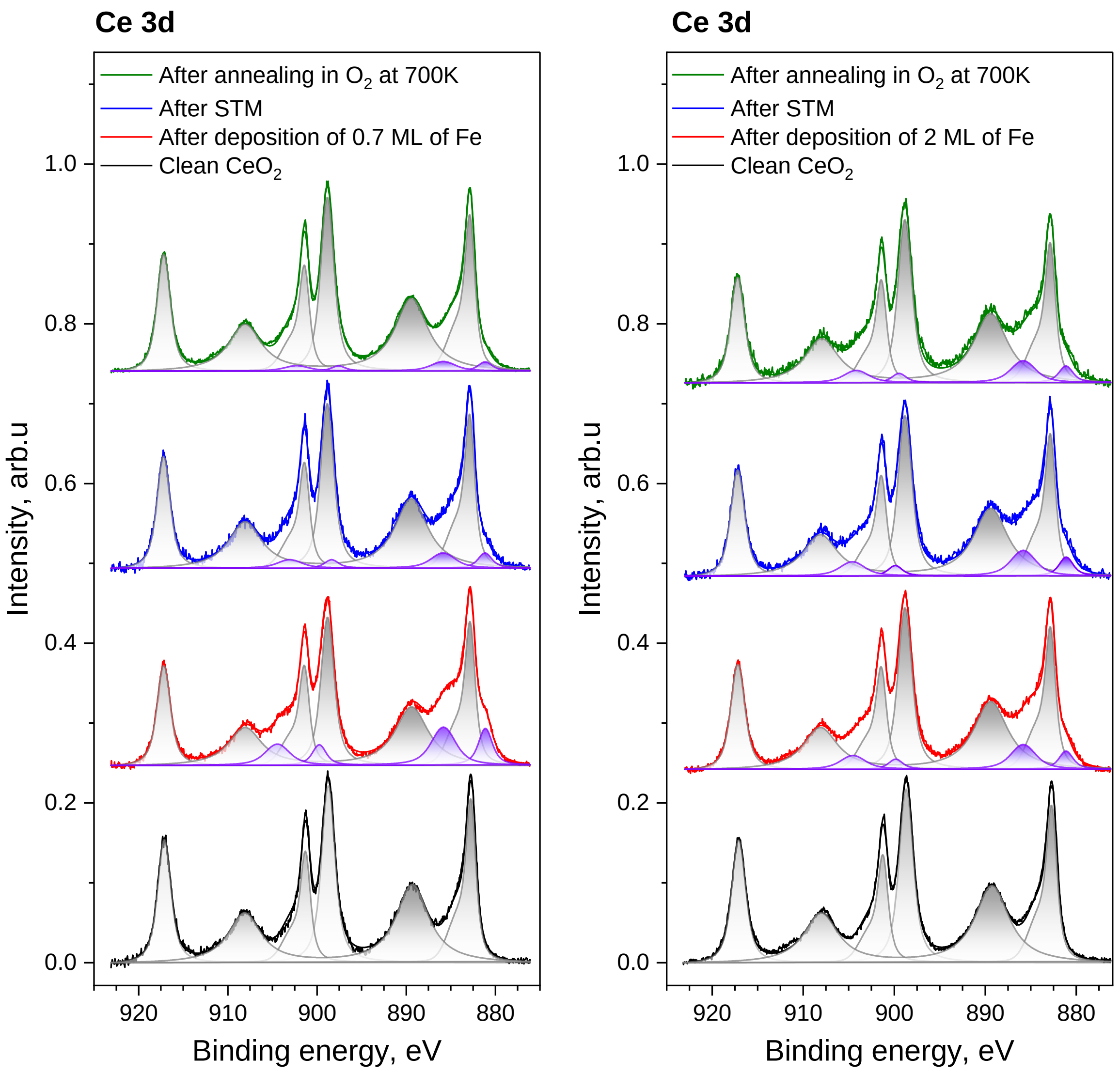

Figure S5. The in-situ XPS Ce 3d core level spectra from the clean $CeO_2(111)$ surface as well as after the deposition of 0.7 ML (left) and 2 ML of iron (right), after the STM experiment and after annealing in 1×10^{-8} mbar of O_2. The spectra were acquired with Al Kα radiation (1486.6 eV) and were normalized to the same area. Three Ce^{4+}-related doublets are marked in gray and two Ce^{3+}-related doublets are marked in violet. Deconvolution of the Ce 3d spectra was performed following the procedure described in ref. [18].

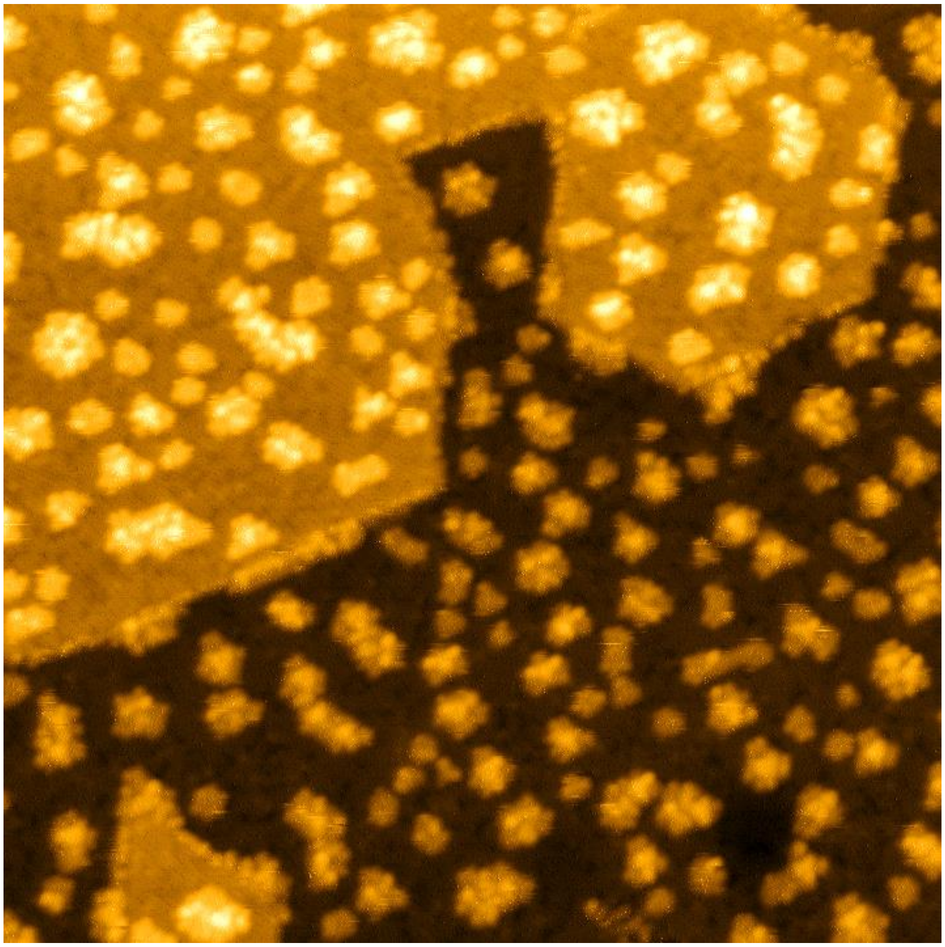

Figure S6. STM image of the $FeO_x/CeO_2(111)$ surface prepared by deposition of about 0.2 ML of Fe on the $CeO_2(111)$ substrate at 300 K and annealing at 700 K in UHV. Surface area 70 × 70 nm^2, $U_{bias} \approx 3$ V, $I_t \approx 10$ pA.

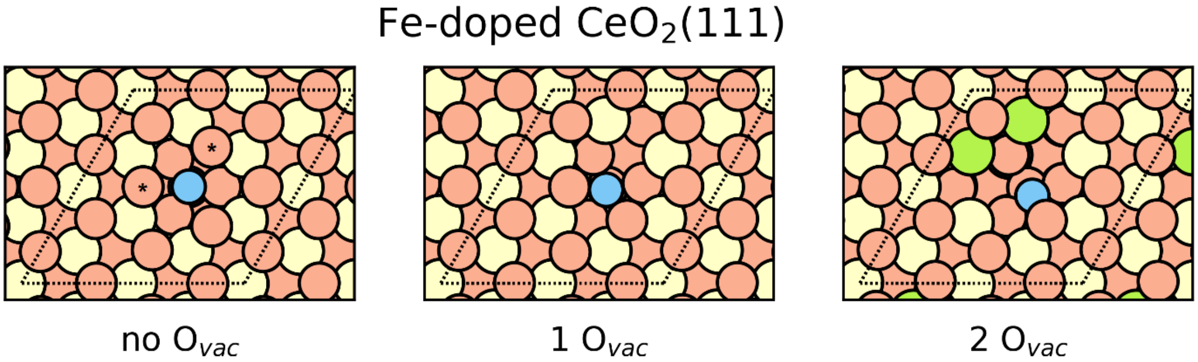

Figure S7: Relaxed structures of the models used to represent the fully oxidized and reduced Fe-doped CeO_2(111) surface. An outermost Ce atom has been replaced by a Fe atom in the $CeO_2(2\sqrt{3}\times2\sqrt{3})$ slab model, leading to 1/12 coverage of Fe. To form oxygen vacancies, O atoms bonded to the Fe atom (indicated with asterisks in the left image) have been removed from the outermost O layer. Blue, beige, green, and orange circles correspond to Fe, Ce^{4+}, Ce^{3+}, and O ceria atoms, respectively.

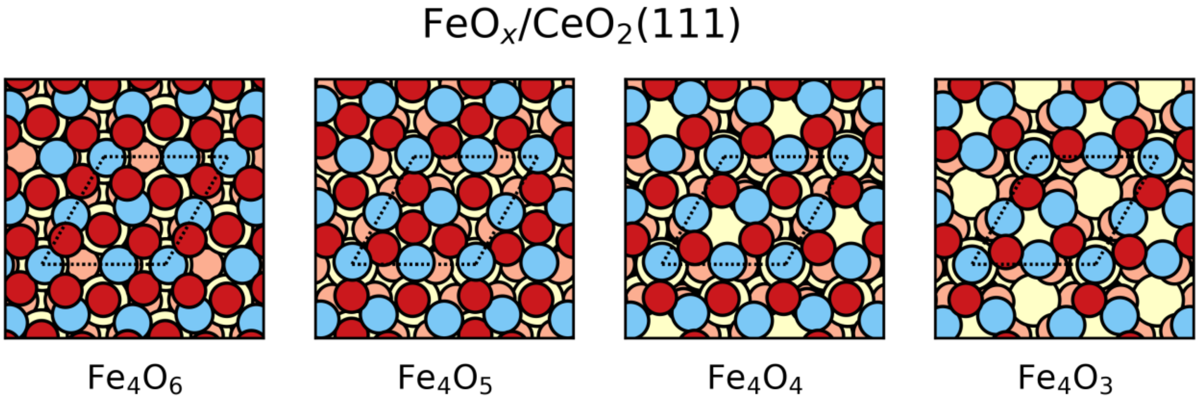

Figure S8: Relaxed structures of the models used to calculate $E_f(O_{vac})$ in CeO_2(111)-supported 2D FeO_x. Blue, red, beige and orange circles correspond to Fe, O atoms in FeO and Ce, O atoms in CeO_2, respectively.

References:

(1) Ge, C.; Yu, Y.; An, D.; Tong, Q.; Tang, C.; Gao, F.; Sun, J.; Dong, L. Surface Configuration Modulation for FeO-CeO_2/γ-Al_2O_3 Catalysts and Its Influence in CO Oxidation. *Journal of Catalysis* **2020**, *386*, 139–150. https://doi.org/10.1016/j.jcat.2020.04.005.

(2) Grosvenor, A. P.; Kobe, B. A.; Biesinger, M. C.; McIntyre, N. S. Investigation of Multiplet Splitting of Fe 2*p* XPS Spectra and Bonding in Iron Compounds. *Surf. Interface Anal.* **2004**, *36* (12), 1564–1574. https://doi.org/10.1002/sia.1984.

(3) Biesinger, M. C.; Payne, B. P.; Grosvenor, A. P.; Lau, L. W. M.; Gerson, A. R.; Smart, R. St. C. Resolving Surface Chemical States in XPS Analysis of First Row Transition Metals, Oxides and Hydroxides: Cr, Mn, Fe, Co and Ni. *Applied Surface Science* **2011**, *257* (7), 2717–2730. https://doi.org/10.1016/j.apsusc.2010.10.051.

(4) Pauly, N.; Yubero, F.; Espinós, J. P.; Tougaard, S. XPS Primary Excitation Spectra of Zn 2*p*, Fe 2*p*, and Ce 3*d* from ZnO, α-Fe_3O_2, and CeO_2. *Surface Interface Analysis* **2018**, *51* (3), 353–360. https://doi.org/10.1002/sia.6587.

(5) Fujii, T.; De Groot, F. M. F.; Sawatzky, G. A.; Voogt, F. C.; Hibma, T.; Okada, K. *In Situ* XPS Analysis of Various Iron Oxide Films Grown by NO_2-Assisted Molecular-Beam Epitaxy. *Phys. Rev. B* **1999**, *59* (4), 3195–3202. https://doi.org/10.1103/PhysRevB.59.3195.

(6) Droubay, T.; Chambers, S. A. Surface-Sensitive Fe 2*p* Photoemission Spectra for α-Fe_2O (0001): The Influence of Symmetry and Crystal-Field Strength. *Phys. Rev. B* **2001**, *64* (20), 205414. https://doi.org/10.1103/PhysRevB.64.205414.

(7) Wang, W.; Tang, B.; Wu, S.; Gao, Z.; Ju, B.; Teng, X.; Zhang, S. Controllable 5-Sulfosalicylic Acid Assisted Solvothermal Synthesis of Monodispersed Superparamagnetic Fe_3O_4 Nanoclusters with Tunable Size. *Journal of Magnetism and Magnetic Materials* **2017**, *423*, 111–117. https://doi.org/10.1016/j.jmmm.2016.09.089.

(8) Bravo Sanchez, M.; Huerta-Ruelas, J. A.; Cabrera-German, D.; Herrera-Gomez, A. Composition Assessment of Ferric Oxide by Accurate Peak Fitting of the Fe 2*p* Photoemission Spectrum: Composition of Ferric Oxide by Peak Fitting the Fe 2*p* Spectrum. *Surf. Interface Anal.* **2017**, *49* (4), 253–260. https://doi.org/10.1002/sia.6124.

(9) Chandra, S.; Das, R.; Kalappattil, V.; Eggers, T.; Harnagea, C.; Nechache, R.; Phan, M.-H.; Rosei, F.; Srikanth, H. Epitaxial Magnetite Nanorods with Enhanced Room Temperature Magnetic Anisotropy. *Nanoscale* **2017**, *9* (23), 7858–7867. https://doi.org/10.1039/C7NR01541K.

(10) Hou, Z.; Yan, P.; Sun, B.; Elshekh, H.; Yan, B. An Excellent Soft Magnetic Fe/Fe_3O_4-FeSiAl Composite with High Permeability and Low Core Loss. *Results in Physics* **2019**, *14*, 102498. https://doi.org/10.1016/j.rinp.2019.102498.

(11) Li, B.; Rong, T.; Du, X.; Shen, Y.; Shen, Y. Preparation of Fe_3O_4 Particles with Unique Structures from Nickel Slag for Enhancing Microwave Absorption Properties. *Ceramics International* **2021**, *47* (13), 18848–18857. https://doi.org/10.1016/j.ceramint.2021.03.224.

(12) Wang, Y.; Wang, L. L.; Sun, C. Q. The 2p3/2 Binding Energy Shift of Fe Surface and Fe Nanoparticles. *Chemical Physics Letters* **2009**, *480* (4–6), 243–246. https://doi.org/10.1016/j.cplett.2009.09.017.

(13) Temesghen, W.; Sherwood, P. Analytical Utility of Valence Band X-Ray Photoelectron Spectroscopy of Iron and Its Oxides, with Spectral Interpretation by Cluster and Band Structure Calculations. *Anal Bioanal Chem* **2002**, *373* (7), 601–608. https://doi.org/10.1007/s00216-002-1362-3.

(14) He, Q.; Shi, H.; Wang, Y.; Cao, L.; Gu, X.; Wu, J.; Hong, G.; Li, M. High-Dose X-Ray Radiation Induced MgO Degradation and Breakdown in Spin Transfer Torque Magnetic Tunnel Junctions. *Sci Rep* **2022**, *12* (1), 18620. https://doi.org/10.1038/s41598-022-19342-x.

(15) Yamashita, T.; Hayes, P. Analysis of XPS Spectra of Fe^{2+} and Fe^{3+} Ions in Oxide Materials. *Applied Surface Science* **2008**, *254* (8), 2441–2449. https://doi.org/10.1016/j.apsusc.2007.09.063.

(16) Bhattacharjee, S.; Mazumder, N.; Mondal, S.; Panigrahi, K.; Banerjee, A.; Das, D.; Sarkar, S.; Roy, D.; Chattopadhyay, K. K. Size-Modulation of Functionalized Fe_3O_4: Nanoscopic Customization to Devise Resolute Piezoelectric Nanocomposites. *Dalton Trans.* **2020**, *49* (23), 7872–7890. https://doi.org/10.1039/D0DT01167C.

(17) Ertl, G.; Wandelt, K. Electron Spectroscopic Studies of Clean and Oxidized Iron. *Surface Science* **1975**, *50* (2), 479–492. https://doi.org/10.1016/0039-6028(75)90038-2.

(18) Skála, T.; Šutara, F.; Škoda, M.; Prince, K. C.; Matolín, V. Palladium Interaction with CeO_2 , Sn–Ce–O and Ga–Ce–O Layers. *J. Phys.: Condens. Matter* **2009**, *21* (5), 055005. https://doi.org/10.1088/0953-8984/21/5/055005.
